# Nanomaterial-Doped Xerogels for Biosensing Measurements of Xanthine in Clinical and Industrial Applications

**DOI:** 10.3390/gels9060437

**Published:** 2023-05-25

**Authors:** Quang Minh Dang, Ann H. Wemple, Michael C. Leopold

**Affiliations:** Department of Chemistry, Gottwald Center for the Sciences, University of Richmond, Richmond, VA 23173, USA; harry.dang@richmond.edu (Q.M.D.); holly.wemple@richmond.edu (A.H.W.)

**Keywords:** first generation biosensor, xanthine, gold nanoparticle, monolayer-protected clusters, xerogel, layer-by-layer assembly

## Abstract

First-generation amperometric xanthine (XAN) biosensors, assembled via layer-by-layer methodology and featuring xerogels doped with gold nanoparticles (Au-NPs), were the focus of this study and involved both fundamental exploration of the materials as well as demonstrated usage of the biosensor in both clinical (disease diagnosis) and industrial (meat freshness) applications. Voltammetry and amperometry were used to characterize and optimize the functional layers of the biosensor design including a xerogel with and without embedded xanthine oxidase enzyme (XOx) and an outer, semi-permeable blended polyurethane (PU) layer. Specifically, the porosity/hydrophobicity of xerogels formed from silane precursors and different compositions of PU were examined for their impact on the XAN biosensing mechanism. Doping the xerogel layer with different alkanethiol protected Au-NPs was demonstrated as an effective means for enhancing biosensor performance including improved sensitivity, linear range, and response time, as well as stabilizing XAN sensitivity and discrimination against common interferent species (selectivity) over time—all attributes matching or exceeding most other reported XAN sensors. Part of the study focuses on deconvoluting the amperometric signal generated by the biosensor and determining the contribution from all of the possible electroactive species involved in natural purine metabolism (e.g., uric acid, hypoxanthine) as an important part of designing XAN sensors (schemes amenable to miniaturization, portability, or low production cost). Effective XAN sensors remain relevant as potential tools for both early diagnosis of diseases as well as for industrial food monitoring.

## 1. Introduction and Background

Enzymatic-based electrochemical biosensors [1,2,3,4] continue to be at the forefront of potential tools being developed for the detection and monitoring of target molecules with relevance to clinical [5], forensic [6], environmental [7,8], and agricultural/food applications [6,9]. Endowed with natural selectivity from enzyme incorporation, electrochemical biosensors of this nature offer a range of properties that are attractive for sensor development such as inexpensive materials/operation, well-understood mechanisms, and portability for on-site diagnostic abilities. These abilities include medical point-of-care (POC) testing, field measurements, and crime-scene preliminary screening tests [10,11,12]. In recent years, the mobility of electrochemical sensors has been a motivating factor for significant research activity aiming to study and produce wearable sensors capable of continuous or on-demand measurement of health-related analytes (e.g., wearable glucose detection patches for diabetic patients) [11,13,14,15]. A major facilitating factor of this research focus is the amenability of electrochemical sensors toward miniaturization and the strategy to engineer sensor design to microneedles or small wires for *in vitro*, POC measurements [11,12] or implantable, *in vivo* sensory devices [16,17,18].

The desire to miniaturize electrochemical sensors for certain applications has consequently created a need to enhance the performance of biosensing schemes, most notably amplification of signal. One strategy toward achieving this enhancement is the incorporation of nanomaterials (NMs) into sensing schemes [19,20,21] which often employ mesoporous [22] or nanostructured scaffolds for the enzymes at the electrode [19,20,22,23,24] and/or utilizing nanoparticles (NPs) (e.g., carbon nanotubes (CNTs) [25,26], metallic clusters [18] as a functional component within the composite films. These approaches have been investigated for environmental [27], clinical [24,28], and forensic-related sensors [29,30]. A classic subset of this body of work is the incorporation of NMs within first generation amperometric biosensors, also known as indirect biosensors, in which immobilized enzyme at an electrode reacts with targeted substrate in the presence of oxygen to produce hydrogen peroxide (H_2_O_2_) that is subsequently oxidized at the electrode interface to produce an analytical signal [2,3,4]. While some studies of this nature employ NMs directly at the electrode interface, others disperse NPs within the same matrix that is used to immobilize the enzymes [31].

Sol-gel chemistry has been explored for decades as a means of encapsulating molecules within a porous, gelatinous matrix at an electrode [32]. With their high surface area, porosity, ease-of-preparation/application, and established biocompatibility, polymeric xerogels represent a common formulation of sol-gels utilized in biomedical, drug delivery, and sensors research [33,34,35]. An intrinsically simple process, xerogels are formed from solutions of silane precursor molecules where evaporation of solvent proceeds under ambient conditions. As biomolecules can be included in these mixtures where they become immobilized in the forming cross-linked gel while still preserving their bio-functionality, the three-dimensional xerogel remains a prominent material used in biosensor studies, particularly for clinical/biomedical applications [33,34,35]. Xerogels offer several advantageous properties for biosensing including mild synthetic conditions that preserve the function/structure of embedded enzymes, chemical inertness, physical rigidity, negligible swelling in aqueous solutions, thermal/photochemical stability, and synthetically tunable porosity, the latter being a property of particular interest [36]. Challenges of using xerogels in this capacity include that the films can be (a) prone to enzyme leakage, (b) restrict entry of the targeted substrate in the film, and (c) introduce diffusional barriers that affect H_2_O_2_ diffusion through the layer to the electrode—the key to signal generation in 1st generation biosensing designs [37,38].

For several years, our research group has focused on the incorporation of NMs into xerogel-based, first generation enzymatic biosensors as a strategy to enhance biosensor signal for clinical applications. We have explored both the direct modification of the electrode interface with platinum NPs [39] or CNTs [26,40] as well as dispersing gold NPs throughout the xerogels scaffold housing the enzyme within the scheme [41]. Both strategies were executed within layer-by-layer (LbL) biosensor construction schemes. In this manner, we were able to demonstrate NM-enhanced signal amplification for a number of biosensors targeting clinically relevant disease/conditions including for the detection of glucose (diabetes monitoring), uric acid (gout, preeclampsia risk) [39], sarcosine (prostate cancer diagnostic) [40], lactate (sepsis diagnosis) [18], and galactose (infant galactosemia) [26]. The body of work established a versatile and adaptable strategy, including methodology and materials, toward a range of specific targets and sensing platforms, including successful miniaturization to needle-like electrodes and functionality in bodily fluids relevant to the diagnostic needs (e.g., blood, urine) [18].

Xanthine (XAN) (3,7-dihydro-purine-2,6-dione, Supporting Information, Scheme S1) represents a more complex sensing target with a complicated metabolic mechanism that is relevant to *both* clinical and industrial applications [38]. The XAN molecule is a key component of the purine metabolism cycle, a by-product generated during adenosine triphosphate (ATP) degradation in humans and animals. The metabolic cycle, shown in Scheme S1, shows that both XAN and hypoxanthine (HXAN) are converted to uric acid (UA) and/or H_2_O_2_ via enzymatic reaction with xanthine oxidase (XOx), a molybdoflavin enzyme. As the key enzyme in purine metabolism, XOx is broadly distributed within the tissue of mammals [42,43]. Clinically, XAN levels are an early indicator of abnormal purine metabolism that can result from a number of disease conditions [44]. Normal levels of XAN in blood serum are expected to be low (~0.6 mg/L), some 100-fold less than UA blood serum levels. Elevated levels can be indicative of Lesch–Nyhan Syndrome, a serious condition occurring in males and characterized by neurological and behavioral abnormalities. If purine metabolism is suppressed, XAN build-up in blood serum and muscle can be implicated in the genetic disease xanthinuria as well as urinary tract infection/disease, kidney stones, and renal failure [38]. XAN concentration in urine is typically low as a consequence of healthy metabolism which efficiently converts it to UA (Scheme S1). With low solubility, XAN is rapidly removed via the renal system at a rate ten times that of UA filtering, the latter present in urine at significantly higher concentrations (~2–8 mM). As such, detection of XAN in urine above normal levels (~40–160 μM) can serve as an early diagnostic for many of these disease/conditions [38].

Within the food/agriculture industry, highly portable sensors operational by non-experts in manufacturing plants are viewed as effective tools for monitoring the freshness of meat. Despite this need, there are a limited number of studies describing robust systems toward that specific application [8,9,45]. XAN levels can serve as an early indicator of meat freshness before the food product exhibits signs of spoilage (i.e., discoloration, bacterial growth, odor) [42,43]. With the storage of meat, continual ATP degradation leads to HXAN production and subsequent transformation to XAN, ultimately forming UA. Indeed, there are a number of sensors developed for food freshness measurements that target HXAN as the indicator species, including a recent report by Wang et al. utilizing a bienzymatic scheme for a colorimetric sensor [46]. After slaughter, XAN levels continuously increase in meat tissue over time and is slowed only by refrigeration/freezing. As such, the level of XAN in meat samples can indicate the degree of spoilage of the food. High levels of XAN in meat are correlated with the foul stench of spoiled meat [45]. Benvidi et al. used an enzyme-based potential-sweeping electrochemical sensor, supplemented with CNTs, to show XAN concentration in salmon meat increased to around 25 μM in 25 days [47]. Similarly, Dervisevic et al. developed an amperometric biosensor, augmented with gold NPs, to record steady increases in XAN concentration from approximately 2.5 μM (5 days) to around 20, 27, and 32 μM (25 days) for beef, chicken, and fish samples, respectively [43]. Given their inherent portability and low cost, development of effective electrochemical XAN sensors for industrial use remains of high interest.

The detection of XAN via a first generation biosensing scheme represents a formidable scientific challenge for a number of reasons. First and foremost, the dual need to detect XAN in clinical and industrial settings, requires schemes to be engineerable for the specific application, including appropriate limits of detection (sensitivity) and/or concentration ranges. Ideally, one sensor design could be calibrated for both applications—low concentrations up to a maximum of 50 μM for meat spoilage and a wider linear range for clinical monitoring of XAN levels. Second, while XAN undergoes an enzymatic reaction with XOx like many other first generation biosensing schemes, it is a significantly more complex because of the products of that specific reaction [43,48]. As shown below, the XOx-catalyzed reaction of XAN Rxn. (1) produces UA and/or H_2_O_2_, both of which can be oxidized at an electrode interface (Rxns. (2a) and (2b)) depending on the applied potential:

(1) $$\mathrm{XAN}+{\;O}_{2}\;\overset{\mathrm{XOx}}{\to }\;\mathrm{UA}\;+{\;H}_{2}O_{2}$$

(2a) $$H_{2}O_{2}\;\overset{\;}{\to }2H^{+}+O_{2}+2e^{-}$$

(2b) $$\mathrm{UA}\;\overset{\;}{\to }{\mathrm{UA}}^{2+}+2H^{+}+2e^{-}$$

Unlike many previously developed first generation enzymatic biosensors, [18,26,39,40,41] the substrate/reactant [1] itself, XAN in this case, is also electroactive. Additionally, XOx can also react with HXAN as a substrate to produce the same products as well (Scheme S1). The XOx enzyme has been linked to significant production of reactive oxygen species (ROS), making XAN presence an indicator of vascular inflammation under ischemic or hypoxic conditions where H_2_O_2_ production will be the more dominant (i.e., up to 90%) [49]. In this respect, the development of any electrochemical XAN biosensor must consider both oxygen dependence [49,50] and applied potential dependence [18,48,51,52] to fully understand the factors generating the observed signal. Some XAN biosensor studies do not address the potential contributions of electroactive species other than XAN even though high positive potentials are being employed during analysis [51,53,54,55,56].

High quality review articles are available [38,42] that highlight the range of different electrochemical sensors and biosensors that have been developed for XAN detection, including reports of non-enzymatic sensors [57], sensing schemes based on potential sweep techniques (e.g., differential pulse or cyclic voltammetry) [47,52,57,58], as well as second/third generation amperometric sensing schemes [44]. Herein, we focus specifically on the body of work employing traditional, first-generation XAN biosensing schemes where XOx, immobilized at the electrode interface, produces an electroactive species whose subsequent oxidation [51,59] or reduction [60,61] indirectly reports the presence XAN in solution. In particular, our interest remains biosensing schemes that incorporate NMs, many of which are highlighted in a recent, review articles [24,42]. Just in the past few years, Khan et al. reported the use of functionalized gold NPs within a polymer matrix to detect XAN in food samples [52] while Benvidi and coworkers utilized an electrode modified with multi-walled CNTs that immobilized XOx for XAN detection in meat [47]. In 2019, Sayer and coworkers reported electrochemical XAN detection using electrodes modified with polypyrrole films housing Ag-doped ZnO NPs [56]. Even with the work accomplished in this area, a highly portable electrochemical sensor, adaptable to both industrial and clinical application, and the ability to deconvolute the signal remains a desirable scientific goal.

In this study, various aspects of a xerogel-based, LbL-assembled, first-generation xanthine amperometric biosensor are systematically explored. Each layer of the modified electrode is electrochemically analyzed and optimized before being combined into a fully functional XAN biosensor, including xerogel layering with either hydrophilic or hydrophobic character. Additionally, the incorporation of an NP network within the XOx enzyme encapsulating xerogel layer is explored as a signal enhancement strategy. The analytical performance of the full biosensor is then established by characterizing its XAN sensitivity, selectivity against common interferent species, response time, and stability. Because the XOx enzymatic reaction used in the biosensor involves multiple electroactive species, part of this study is dedicated to gaining a full understanding of the oxidative current that is observed during operation. Finally, the study involves the demonstrated use of the biosensor for clinical and industrial applications.

## 2. Results and Discussion

**Figure 1:**
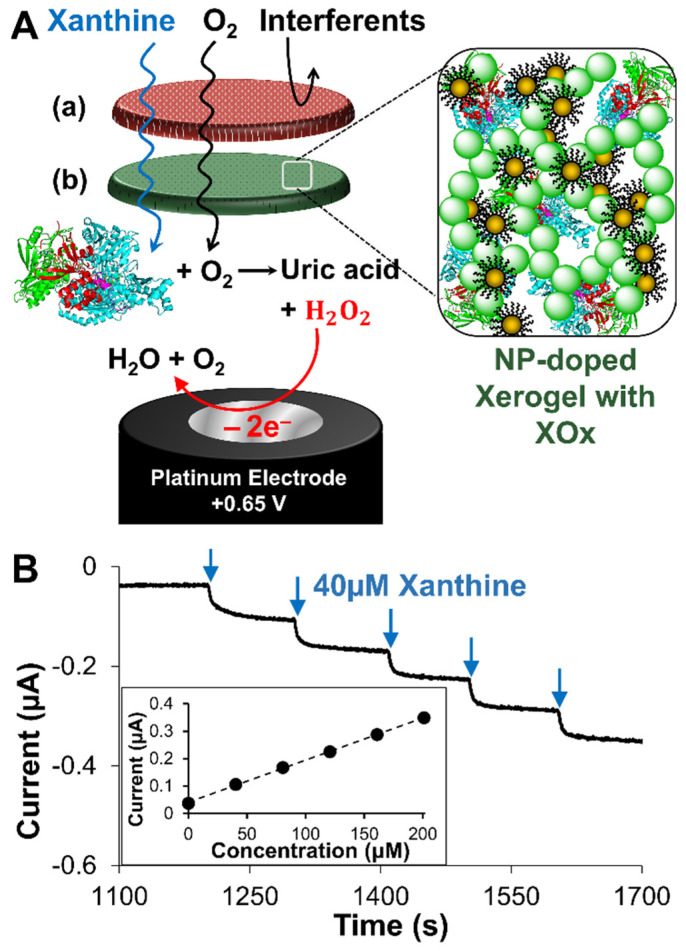
(**A**) Schematic of LbL constructed xerogel-based XAN biosensor featuring (**a**) a polyurethane outer membrane and (**b**) a xerogel layer encapsulating XOx that can be doped with a network of gold NPs all modifying a Pt electrode. The electrode is held at a potential to oxidize the product(s) of the enzymatic reaction, which generates a current signal that indicates XAN presence in solution. (**B**) Illustrative examples of a typical amperometric It curve generated with successive injections of standardized XAN showing the characteristic stair-step responses that can be translated to a calibration curve (Fig. 1B, inset, *n* = 1 for illustration). Note: Notation for the assembled biosensors in this study uses the following format: **Pt/HMTES (XOx) + Au NPs/PU (75:25)** to indicate a Pt electrode modified with a single xerogel layer that encloses XOx and is doped with Au-NPs, all of which is capped with a PU layer comprised of 75:25 HPU:TPU (*w*/*w*) blended composition.

The modified electrode serving as the xanthine (XAN) biosensor is shown in Figure 1A and features an LbL assembly of several layers that each perform a specific function. These layers include the following: (1) a xerogel layer for encapsulating active xanthine oxidase (XOx) enzyme which converts XAN substrate into the hydrogen peroxide (H_2_O_2_) byproduct that is then ultimately oxidized at the electrode interface to produce the signal; (2) an undoped (i.e., no XOx) diffusional layer of xerogel that limits both the diffusional approach of XAN to avoid Michaelis-Menten kinetic effects and attenuates diffusional loss of H_2_O_2_ oxidation signal from the electrode; and (3) a capping layer of blended polyurethanes (PU) that adds stability, robustness, and assists with selectivity. As with traditional first-generation biosensing schemes, the electrode can be held at an oxidizing potential for H_2_O_2_ during sequential injections of substrate (e.g., XAN) to produce a stair-step amperometric response (I-t curve) that is then translated to a calibration curve (Figure 1B). Similar schemes have been successfully developed for a number of different analyte species (e.g., glucose, uric acid, sarcosine) and have established that each of these layers needs to be optimized toward a specific targeted molecule to create an effective biosensor [62,63]. Herein, we show the optimization of this LbL approach for the detection of XAN for both industrial and clinical application. As previously mentioned, the challenge of this study is the deconvolution and understanding of how multiple electroactive components within the metabolic mechanism, including XAN, HXAN, uric acid (UA), and H_2_O_2_ can affect the performance of the sensor, as well as signal enhancement strategies using an NP network for specific applications.

### 2.1. Layer-by-Layer Optimization

#### 2.1.1. Outer Selective Polyurethane (PU) Layer

As previously mentioned, the PU layer acts as a semi-permeable membrane that both controls the diffusional approach of XAN and oxygen into the film assembly but also provides stability, robustness, and partial selectivity via its hydrophobic/hydrophilic properties. In this study, different blends of hydrophilic and hydrophobic PUs, known as HPU and TPU, respectively, were evaluated for XAN biosensing. Ideally, based on the reaction mechanism and electrode modification (Figure 1A), an optimal PU layer should allow for XAN and O_2_ entry into and through the outer layer while also at least partially prohibiting entry of other interferent species. Similarly, an ideal capping layer would also prohibit significant leakage of H_2_O_2_ produced in the enzymatic reaction, though this is often better evaluated with all the layers present.

**Figure 2:**
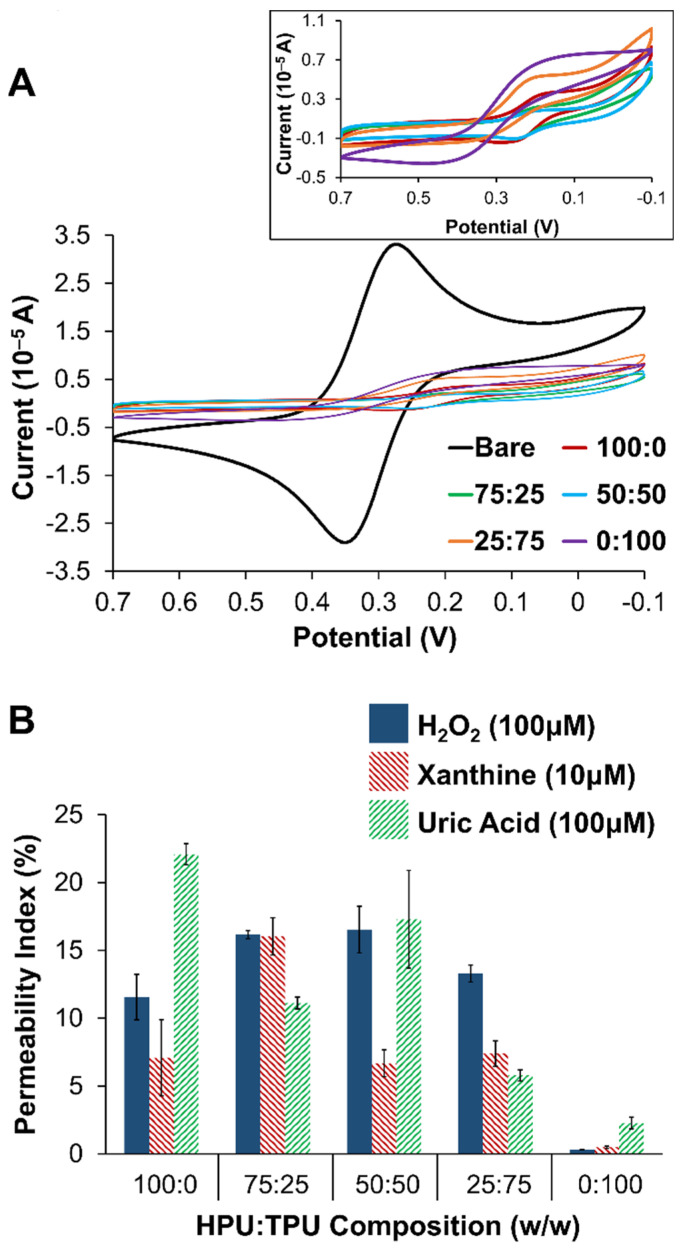
(**A**) Cyclic voltammetry (100 mV/s) of 5 mM potassium ferricyanide (0.5 M KCl) at PU layers of different compositions (ratios of HPU:TPU) compared to the same voltammetry at a bare/unmodified Pt electrode, including an expanded view of the voltammetry of all PU layers without the unmodified electrode (**inset**). (**B**) Permeability indices (PI) for H_2_O_2_, XAN, and UA injections of different compositions of the PU layer (i.e., different ratios of HPU:TPU). **Note:** Error bars reflect standard error.

Electrochemical analysis of the different layers of the biosensing scheme, including the PU layer, was conducted with two methods. First, to confirm the presence and porosity of layers, cyclic voltammetry (CV) of a probe molecule, potassium ferricyanide (FeCN), was collected at each type of blended PU layer. Figure 2A shows a representative example of FeCN probing of the different PU layers in comparison to the same voltammetry at an unmodified (bare) platinum. From the results, it is relatively easy to ascertain that the main effect of any of the PU layers was to act as a substantial barrier to diffusional species. Figure 2A (inset) expands the voltammetry without the bare electrode response to illustrate the subtle differences between the different PU blends. Permeability indices (PIs) (Section 2.3) were also measured for both XAN and H_2_O_2_ at all the different PU as well as UA given that it is likely to be present from the mechanism (Supporting Information, Scheme S1) and electroactive. Figure 2B summarizes the PI for the different PU layers. For the purposes of developing this particular biosensor, entry of the XAN was the priority and the 75:25 HPU:TPU PU blend was selected as the outermost layer.

#### 2.1.2. Xerogel Optimization—Silane Precursors, Aging, Enzyme Loading, and Multi-Layers

**Figure 3:**
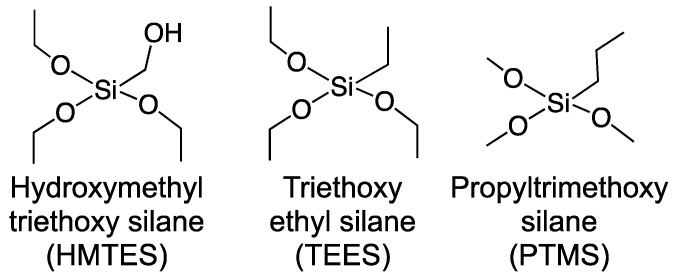
Silane precursor molecules used to form cross-liked xerogels.

A variety of silane precursor molecules have been used to form over the years [32]. Prior work in our lab suggested that for the encapsulation of XOx within a first generation biosensing scheme, hydroxymethytriethoxy silane (HMTES), triethoxyethyl silane (TEES), and propyltrimethoxy silane (PTMS) were promising. Shown in Figure 3, these silane precursor molecules not only offer side chains of varying hydrophobicity (R = hydroxyl, ethyl, propyl), but have been previously demonstrated in biosensing schemes, including those targeting uric acid [63], and yielding promising preliminary results (not shown) for XAN.

As described in the Experimental Details section, there are a number of variables to consider when forming xerogels including the presence of ethanol, age, time, humidity, enzyme loading, enhancement doping, as well as the number of xerogel layers—all of which can affect the porosity and ultimately the performance of the biosensor. Prior investigations showed that it was sometimes beneficial to add a second xerogel layer, known as a diffusional xerogel layer (not depicted in Figure 1A), on top of the enzyme-doped layer [63]. Here, again, these individual variables could be assessed using FeCN probing voltammetry, permeability experiments, and ultimately examining the entire ensemble’s response to XAN. Ultimately, experimentation with PTMS as a precursor, regardless of the other variables, was discontinued as it was often found that the XOx enzyme would not dissolve PTMS mixtures. Additionally, permeability data, mentioned below, showed that PTMS-based xerogels were highly porous compared to HMTES and TEES xerogels.

**Figure 4:**
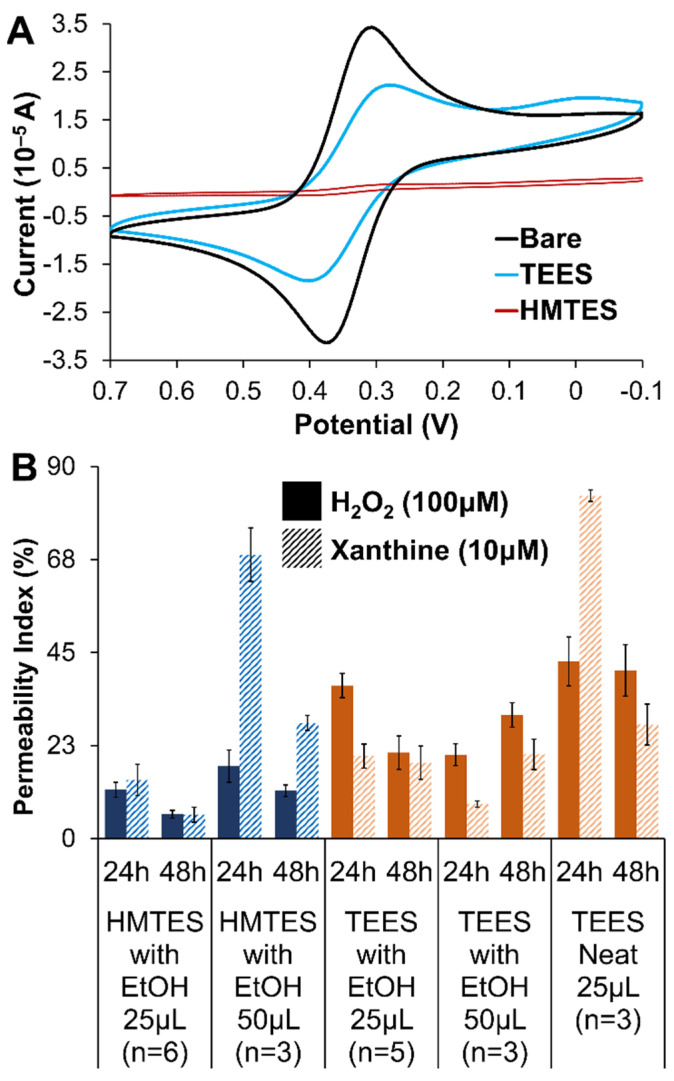
(**A**) Cyclic voltammetry (100 mV/s) of 5 mM potassium ferricyanide (0.5 M KCl) at an HMTES or TEES xerogel layer compared to a bare/unmodified Pt electrode (Note: HMTES and TEES were deposited as a single layer and aged for 48 h in these experiments). (**B**) Permeability indices (PI) for H_2_O_2_ and XAN at xerogels of HMTES (***blue***) and TEES (***orange***) examining the effects of xerogel aging (24 vs. 48 h), xerogel composition (with/without EtOH diluent versus neat) and volume (25 μL versus 50 μL). Note: Error bars reflect standard error.

Electrochemical FeCN probing of single layer HMTES and TEES xerogels (both aged for 48 h) are shown in Figure 4A and display notable differences. Compared to the same experiment at a bare Pt electrode, the voltammetry of FeCN is attenuated at both types of xerogel films but the HMTES xerogel nearly completely blocks the diffusional probe representing a xerogel with a significantly higher degree of cross-linking and lower overall porosity. The blocking effect of both types of films is exasperated if an additional xerogel layer (i.e., a diffusional xerogel layer) is added (Supporting Information, Figure S1). Additionally, when either silane was used within a biosensing scheme featuring two xerogel layers, the XAN response was severely affected, non-existent for the Pt/TEES (XOx)/TEES/PU system and very minimal for Pt/HMTES (XOx)/HMTES/PU system. As such, only systems utilizing one xerogel layer embedded with XOx were explored further.

Similar to the evaluation of the PU layers, permeability measurements were helpful in optimizing the xerogel layers as well. The amperometric response after injecting either H_2_O_2_ or XAN at HMTES or TEES xerogels, aged for either 24 or 48 h and in the presence and absence of ethanol were all evaluated with PI calculations. Optimal xerogels would exhibit sufficient permeability of both XAN and H_2_O_2_ which both have to move through the film to the XOx with H_2_O_2_ then diffusing to the Pt electrode interface (Figure 1A). Additionally, because xerogels continue to cross-link over aging time, it is more ideal if the films are permeable to these species and stable over time (i.e., minimal change and/or increasing permeability over the course of 24–48 h). Figure 4B summarizes the PI measured for all the different xerogel films while additional experiments with PTMS, discontinued in this study, are provided in Supporting Information (Figure S2). Notably, high permeabilities for both XAN and H_2_O_2_ are observed for HMTES with ethanol (50 μL), stabilizing after 48 h of aging. Increasing permeability was observed for the TEES system under the same conditions over the course of 48 h. Considering all the voltammetry and permeability results, it was determined that the study would focus on biosensor assemblies where Pt electrodes were modified with a single layer of either HMTES with ethanol aged for 48 h or TEES with ethanol aged for 24 h before being capped with the PU layer of 75:25 HPU:TPU.

Critical to first generation amperometric biosensing schemes is the amount of enzyme encapsulated in the xerogel layer. Previous studies in our group show that maximizing the enzyme loading within the xerogel typically results in the highest sensitivity [40,63]. This system was consistent with prior findings in that higher loading (12 mg) of the XOx into the xerogel formulation resulted in the highest sensitivity for both types of xerogels. An example of this type of result is provided in Supporting Information (Figure S3).

#### 2.1.3. Nanoparticle Doping of Xerogels

**Figure 5:**
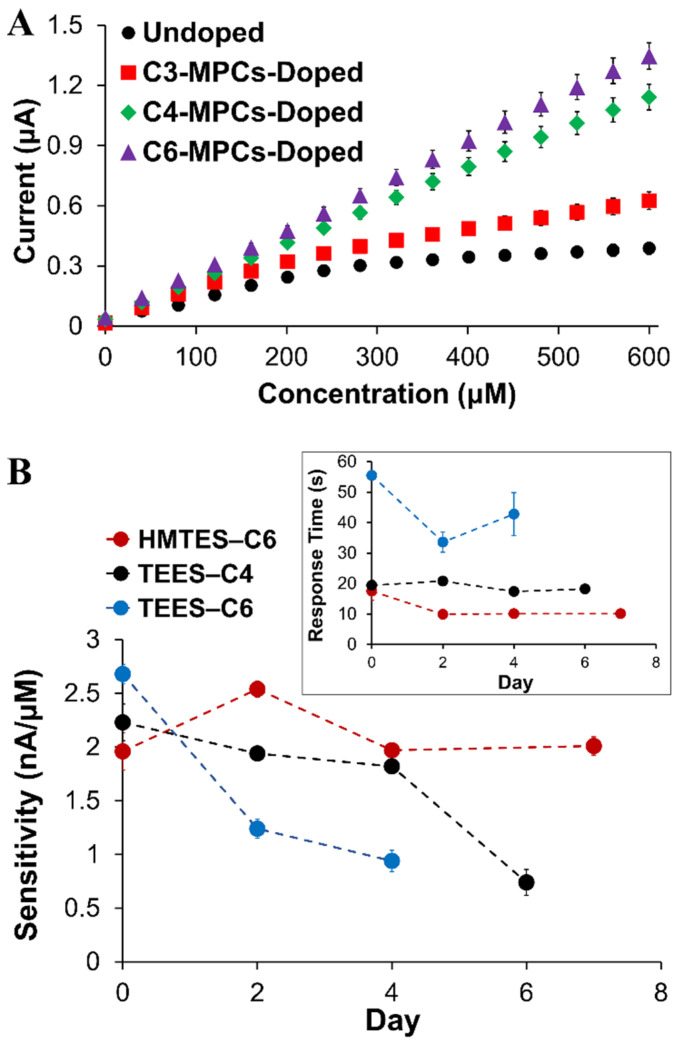
(**A**) Typical calibration curves of HMTES xerogel-based biosensors capped with PU (75:25) in which the XOx-containing xerogel layer is either undoped (*n* = 5) or doped with C3 (*n* = 5), C4 (*n* = 5), or C6-MPCs (*n* = 10). (**B**) Tracking of sensitivity and response time (**inset**) of HMTES (C6-MPCs-doped) and TEES sensors (C4-MPCs-doped) over time. **Notes:** I-t curves (not shown) were collected in PBS (10 mM, pH 7) at a constant potential of +0.65 V; error bars reflect standard error; in some cases, error bars are smaller than marker used for average.

The ability to enhance signal is an important attribute for biosensors, particularly for sensing schemes projected to be applied in complex media (e.g., blood serum, environmental sample digests, and urine). Prior work in our lab showed success in the signal enhancement strategy of incorporating NMs within the modified electrodes, either CNTs [64] or the introduction of an NP network within enzyme-doped xerogel [18,39,41] where signal enhancement and greater sensitivity toward a target analyte was achieved. With the latter strategy, it was previously determined that the NP network serves as a reporting skeleton within the xerogel. Mechanistically, it has been presented that the H_2_O_2_ produced by the enzymatic reaction within the xerogel layer, need only diffuse to the NP network for oxidation rather than to the platinum electrode interface. The MPC network specifically allows for an applied potential within the xerogel as well as an electron transport mechanism to the electrode interface. This mechanism hypothesis, illustrated in the Supporting Information (Scheme S2), was established in a prior study [37]. In this study, an NP network was introduced to both the HMTES and TEES xerogel layer within fully assembled XAN biosensor schemes to assess signal enhancement effects. The NPs used, known as monolayer-protected clusters or MPCs, consisted of a gold core with a stabilizing alkanethiolate periphery where we could alter the chain length of the alkanethiol, using with propanethiol (C3), butanethiol (C4), or hexanethiol (C6) [65]. Figure 5A shows calibration curves derived from amperometric I-t curves of systems incorporating C3, C4, and C6 MPC networks into the HMTES xerogel within the sensing scheme for XAN. Consistent with other biosensing systems where MPC networks are embedded in xerogel layers, three major effects were observed. First, the sensitivity is significantly improved (i.e., higher calibration curve slope) versus film assemblies made with a xerogel layer without MPCs incorporated. The second effect of incorporating the MPC networks is that it results in a notable increase in the linear range of the calibration curve (Figure 5A). Thirdly, faster response times are typically observed for the systems doped with the MPC network. Similar trends were observed for sensors employing the TEES xerogel layer as well where the introduction of the MPC network reduced response times by ~30%. Sensitivity results, as determined from average calibration curves, are shown in Table 1 where the linear range/fit, can also be ascertained from the correlation coefficients (R^2^ values) listed. These results reiterate some of the major findings at this point in the study including improved sensitivity and linear range from incorporating MPC networks and that the use of two xerogel layers versus only one xerogel layer resulted in diminished sensitivity (HMTES) or no amperometric signal (TEES). Calibration curve comparisons for many of these systems within the Table are supplied in Supporting Information (Figures S4–S6). No definitive trends based on the peripheral alkanethiol chain length ligands emerged. That said, we note two specific notable features of the TEES results that seem to be impacted by chain length to some degree. First, response times (t_r-95%_), were generally found to be significantly higher in the TEES systems versus the HMTES system and also showed an increase with increasing alkanethiol chain length of the doped MPCs (Table 1). This trend has been previously observed with other xerogel biosensors we have reported [37]. Second, the TEES systems, while having similar sensitivity as prepared and regardless of MPC doping, tended to show greater instability over time, losing sensitivity (i.e., lower calibration curve slopes) and exhibiting diminished linear range and linearity (i.e., lower R^2^ values) over the course of several days. In contrast, the HMTES systems stabilized at lower response time and sensitivity, exhibiting linear fits (R^2^ values > 0.99) that were persistent over the 7 days of testing. Figure 5B illustrates some of these stability trends for both systems that were derived from corresponding calibration curve results collected over several days and provided in Supporting Information (Figure S7). While not completely understood, this notable difference in the two systems may be related to the general difference in the films hydrophobic character and/or porosity. In general, however, C6-MPC doping of either system resulted in effective sensitivity, though the C4-doped TEES system was slightly more robust over time. Given the totality of these results, it was determined that incorporating a C6 MPC network into either type of xerogel was optimal in terms of enhanced sensitivity and linear range as well as sensor stability over time.

### 2.2. Analytical Performance of Optimized Xanthine Biosensing System

**Figure 6:**
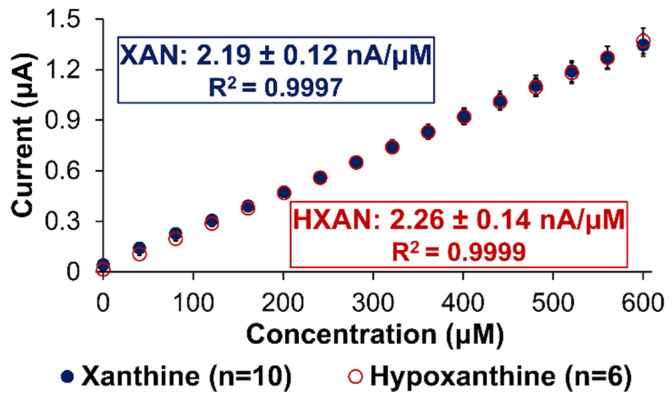
Calibration curves for XAN and HXAN of the optimized Pt/HMTES (XOx) + C6-MPCs/PU (75:25) biosensing scheme at +0.65 V. Note: Error bars reflect standard error; in some cases, error bars are smaller than marker used for average.

The analytical performance of any sensor is determined by assessing a number of key variables including sensitivity, linear range being relevant for targeted systems, limit of detection (LOD), selectivity against interferent species, response time, and stability. In the case of XAN biosensing, it is critical to recognize from the normal metabolism scheme (Scheme S1) that an optimal XAN sensor requires sensitivity to *both* XAN and HXAN since they may both be present and are also both substrates for the XOx enzyme that quickly converts the HXAN to XAN. Thus, our optimized sensors were tested for their responsiveness to both these targets. Figure 6 represents an example of a typical calibration curve for one of our biosensor designs, the Pt/HMTES (XOx) + C6 MPCs/PU(75:25) build, which was tested for both XAN and HXAN sensitivity applying +0.65 V. As seen in the figure, both the XAN and HXAN calibration curves have similar slopes (i.e., sensitivities) from excellent linear regression modeling (i.e., high R^2^ values), and both boast significant linear ranges that easily span the relevant concentrations for XAN detection in blood serum (0.6 mg/L), urine (≤150 μM), and industrial meat spoilage measurements (0–40 μM) [38,42]. For this particular system, a conservative measurement of response time (t_r95%_) was determined for XAN and HXAN as 19.1 (±1.5) and 10.4 (±2.1) s, respectively. The LOD for this HMTES system, using the IUPAC standard (3·σ_blank_/β_1_), was determined as 3.1_(±0.2)_ and 5.2_(±0.1)_ μM with applied potentials of +0.65 and +0.4 V, respectively. A similar calibration curve analysis for the equivalent TEES system is provided in Supporting Information (Figure S8).

**Figure 7:**
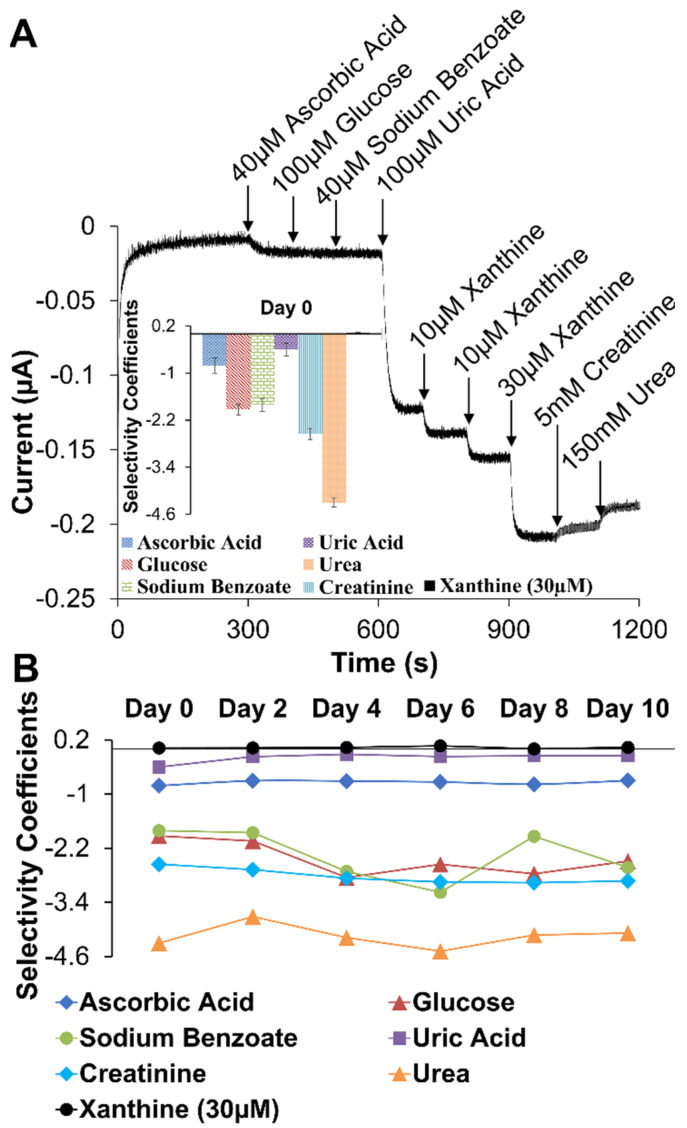
(**A**) Typical amperometric I-t curve generated during interferent testing and (inset) the corresponding selectivity coefficient measurements for common interferents and XAN. (**B**) Tracking of interferent selectivity coefficients for 10 days (*n* = 6). Note: Error bars reflect standard error (**A**) but were removed from (**B**) for clarity.

Selectivity, the discrimination against interferent species, was assessed as in many biosensor reports using an I-t curve generated from sensor assemblies held at a potential during injections of common interferent species identified in the XAN biosensing literature [47,51,56,59,60,66]. In this study, injections of interferent species included testing ascorbic acid (40 μM), glucose (100 μM), sodium benzoate (40 μM), UA (100 μM), and, for potential urine analysis, [40] creatinine (5 mM) and urea (150 mM) were combined with standard injections of XAN at 10 μM and 30 μM—the latter injections to show maintained concentration sensitivity toward XAN. Figure 7A shows the interferent I-t curve for the Pt/HMTES (XOx) + C6 MPCs/PU(75:25) optimized system with only small interferent responses from ascorbic acid, a larger response for UA (discussed below), and expected concentration-dependent responses for XAN injections (i.e., the 30 μM XAN injection response is about 3-fold larger than the 10 μM XAN response). Additionally, there are responses toward both creatinine and urea, though we note that these responses are small in spite of the order of magnitude higher concentration of these interferents. These slight responses toward creatinine and urea do suggest, however, that applying these sensors in a urine analysis may necessitate calibration in a synthetic matrix matching that media.

Relative current responses of interferents and analyte species from an I-t curve can be translated into selectivity coefficients, calculated as described in Experimental Details and displayed in Figure 7A, inset. In this type of analysis, a negative coefficient suggests that an interferent species is effectively discriminated against while a positive coefficient, even a small positive measurement, indicates selectivity for that species. As such, it is clear that this system is effectively discriminating against most of the interferent species while maintaining selectivity for XAN. It is notable, however, that a UA injection that is 10-fold higher in concentration compared to XAN yields a larger response and a smaller negative selectivity coefficient than other interferents. This important observation as well as the electroactivity of UA is discussed further in the next section. The selectivity coefficients were also monitored over the course of 10 days for the Pt/HMTES (XOx) + C6 MPCs/PU(75:25) system as summarized in Figure 7B. The results show sustained, effective discrimination against most interferents as well as continued XAN sensitivity. Individual selectivity coefficient graphs over the course of the 10 days are provided in Supporting Information (Figure S9). A similar selectivity analysis was performed on the TEES system where selectivity results were best for the TEES xerogel layer doped with C4 or C6 MPCs versus C3 MPCs with selectivity coefficients monitored for 1 week (Supporting Information, Figures S10 and S11).

With any biosensing scheme, it is necessary to frame its analytical performance in the context of other sensors reported in the recent literature. Excellent review articles with extensive tables of comparison from the scientific literature are available for both traditional XAN biosensors [38] as well as nanomaterial-assisted XAN biosensing schemes [42]. Additionally, individual studies often include comparison tables [44,54,55] with some of the most recent coming from Pierini et al. [57] comparing different electrochemical methods of XAN biosensors and others studies that contain tables of comparison more narrowly focused on the performance of amperometric biosensors that incorporate NMs, including Dervisevic et al. [43], Sahyar et al. [56], Benvidi et al. [47], and Daizy et al. [52]. In examining this body of work, the xerogel-based XAN biosensors developed in this study are analytically competitive with most other XAN biosensors in multiple parameters. However, given the number of analytical variables that can be compared with each reported system (e.g., sensitivity, LOD, linear range, response time, stability, etc.) and with so many recent reports available that include exhaustive literature comparisons, we have elected to comment on the major attributes as well as less effective properties of our system compared to the bulk of recently reported XAN biosensing systems.

Overall, the optimized biosensors reported in this study could be characterized with well-defined, stair-step current responses, large linear ranges, sufficient sensitivity, effective interferent discrimination, and response times that enable real-time measurements. For example, on average, the Pt/HMTES (XOx) + C6 MPCs/PU(75:25) sensing construct in our study had the highest analytical performance overall. That system tended to exhibit significantly longer linear ranges (0–600 μM) than many of the other sensors found in XAN biosensing literature where some linear ranges were limited to 50 μM [47,57] or even significantly less (<20 μM) [51,56,59,67,68] and, in some cases, notably lower correlation coefficients for calibration curves [57]. The XAN sensitivity (i.e., slope of calibration curve) for our system (~2.2 nA/μM) outperformed some studies that also used NM-based signal enhancement strategies [43,56] but were eclipsed in this category in some cases [47]. In spite of the having lower sensitivity than some other systems, the MPC-doped xerogel biosensors in this study produced I-t curves with well-defined stair-step current response to XAN injections, a result in contrast to other literature reports showing significantly less-defined stepping responses [44,56,60]. Following a similar strategy to our early reporting on doping biosensing interfaces with gold NP networks [41], Dervisevic et al. improved definition of the stair-step current response, increased linear range (from 10–90 μM to 1–200 μM) and doubled sensitivity (from 0.6 to 1.4 nA/μM) as well as LOD and response time by incorporating Au-NPs in a chitosan–polypyrrole matrix at their electrode [43]. We see similar positive impact on these same parameters in this study when the xerogels are doped with an appropriate MPC network. An additional advantage of the reported xerogel biosensing scheme it is relatively equally responsive to both XAN and HXAN (Figure 6), an important aspect since they are likely both simultaneously present and XOx can efficiently convert HXAN to XAN.

One of the more difficult biosensing parameters to compare across the literature is response time. As the tables of comparison contained in either individual studies [43,47,52,56] or review articles [38,42] clearly show, reported response times vary greatly but it is somewhat rare for reports to describe how the response time was actually measured. As in our prior reports [63] and others [60,69] (described in the Experimental Section), a conservative approach to calculating response time is used, determining the t_r95%_, or the time it takes for current signal to reach 95% of its steady state value. In this study, the t_r95%_ values stabilize to ~10 s and 20–50 s for the HMTES and TEES xerogel systems. In either case, these response times are fast enough for real-time measurements of XAN in both clinical and industrial applications.

### 2.3. Signal Differentiation of XAN Biosensor Scheme

**Figure 8:**
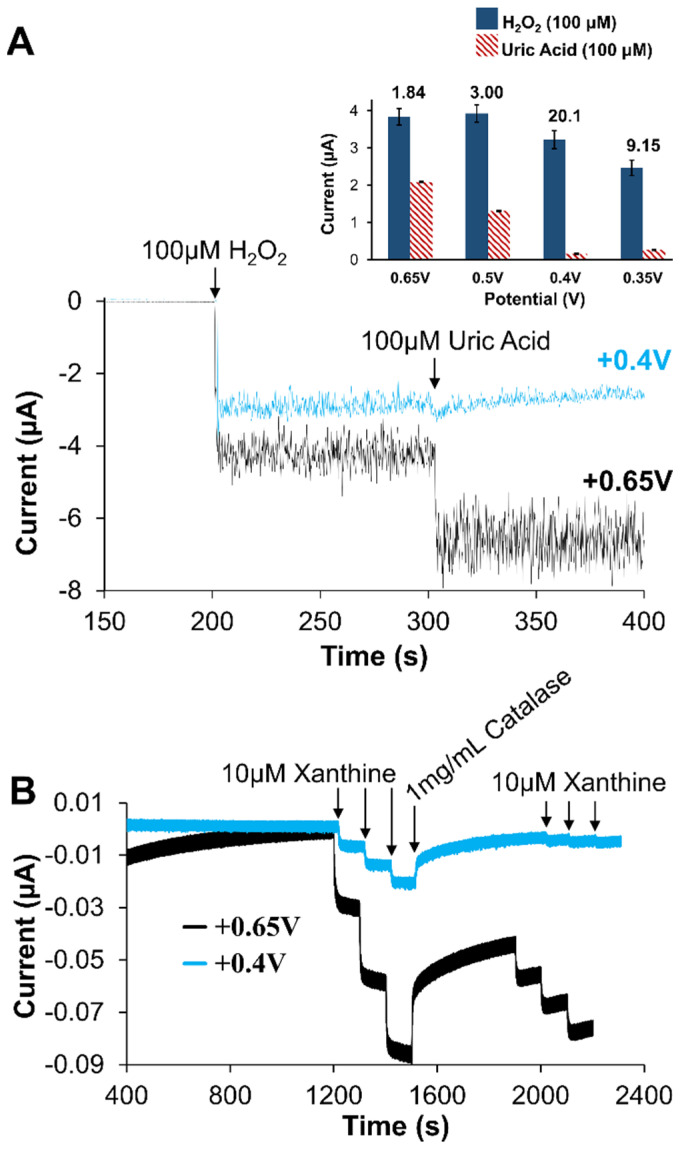
(**A**) Amperometric I-t response to H_2_O_2_ and UA injections (100 μM) at unmodified Pt electrodes held at +0.4 V and +0.65 V; (inset) the current response ratios (H_2_O_2_:UA) derived from I-t curves (Supporting Information, collected at unmodified Pt electrodes held at various potentials (*n* = 3 for each bar). (**B**) Amperometric I-t curves of Pt/HMTES (XOx) + C6-MPCs/PU (75:25) biosensors at +0.65 V versus +0.4 V during standard injections of XAN, interrupted with the addition of catalase, and followed by additional XAN injections. Note: Error bars reflect standard error.

Prior to examining specific application performance of the developed biosensor, a more complete understanding of the observed amperometric was desired—one consistent the metabolic mechanism for XAN. As previously mentioned, the enzymatic mechanism involved with XAN metabolism (Scheme S1) involves multiple electroactive species. While enzyme-based first generation amperometric biosensors are quite common, XAN biosensors represent a more challenging situation since there can be multiple species possibly contributing to the observed current signal. In addition to the XAN and HXAN substrates being electroactive, the products of the XOx enzymatic reaction, UA and H_2_O_2_, are also electroactive. CV experiments of 100 mM solutions of these species show the onset of oxidative current at approximately +0.2 V for UA, +0.3 V for H_2_O_2_ +0.5 V for XAN, +0.85 V for HXAN (vs. Ag/AgCl, satrd. KCl reference) with peak currents being achieved some 150–300 mV later. Examples of these CV results are provided in Supporting Information (Figures S12–S14) and confirm their electroactivity with the oxidizing potentials in agreement with literature reports [52,57]. Some XAN biosensor studies apply relatively high oxidative potentials (≥+0.5 V) but do not address what species may be contributing to the observed current signal [53,55,59,70]. This is particularly true in cases where the constant potential applied is +0.7 V and clearly high enough to oxidize the UA being generated. If more species than just H_2_O_2_ are being generated, accessing the electrode, and being oxidized because of significantly, the signal should exhibit significant potential dependence, particularly with the ratios of UA and H_2_O_2_. Figure 8A summarizes the results of injecting the same concentration of UA and H_2_O_2_ at a bare Pt electrode held at different potentials (+0.65, +0.5, +0.4, and +0.35 V vs. Ag/AgCl; satrd. KCl) reference) and establishes a clear potential dependent signal. Figure 8A (inset) shows an example of the I-t curves during injection of UA and H_2_O_2_ at a bare Pt electrode held at +0.4 V or +0.65 V to further illustrate the importance of the applied potential. Additional experiments yielding I-t curves of this nature are provided in Supporting Information (Figures S15 and S16 and Table S1) and were used to generate the ratios of H_2_O_2_ to UA current for each potential (Figure 8A). The results suggest that if the potential is held, at +0.65 V or higher, the signal is likely the result of *both* UA and H_2_O_2_ generated by the enzymatic reaction. Understanding the UA contribution would be particularly important in cases where there is UA present in biological fluids from sources other than the purine metabolism cycle (Scheme S1) [44,63].

To better understand the contributions to the amperometric signal from UA, the H_2_O_2_ scavenging-enzyme catalase (CAT) was utilized in conjunction with our XAN biosensing schemes [18,26]. The strategy involves the addition of a large amount of CAT during the collection of an I-t curve for the XAN biosensors. If the signal is due only to H_2_O_2_ oxidation, a return to baseline is expected as the H_2_O_2_ is rapidly consumed by the CAT. However, if the observed signal is from a mixture of H_2_O_2_ and UA being oxidized, the CAT will not affect UA and amperometric current should not return to baseline. Figure 8B shows the results of applying that strategy to the optimized system of Pt/HMTES (XOx) + C6 MPC/PU(75:25), held at either +0.65 V or +0.4 V and subjected to successive injections of XAN before the stair-step response is interrupted with a bolus addition of CAT (1 mg/mL). As the I-t curve shows, both potentials yield a stair-step current response to XAN injections, though sensitivity is higher at the +0.65 V sensor. Upon the addition of CAT, it is notable that the sensor held at +0.4 V nearly returns to baseline after the addition of CAT whereas the sensor at +0.65 V is not able to approach baseline. These results suggest that when the XAN biosensor is held at the higher potentials, UA is likely contributing to the observed signal. This point is reiterated again later in the I-t curves (Figure 8B) when XAN injections are made in the presence of (i.e., after the injection of) CAT where, at +0.4 V, injections of the same XAN concentration result in nearly negligible step responses. A clear competition between the CAT and the electrode held at +0.4 V has been established. At the sensor held at +0.65 V, a substantial stepping response returns for the injections in the presence of CAT. Taken collectively, the potential dependent deconvolution of the signal is important to fully understand the functionality of the biosensor design.

### 2.4. Clinical and Industrial Application of XAN Biosensors

**Figure 9:**
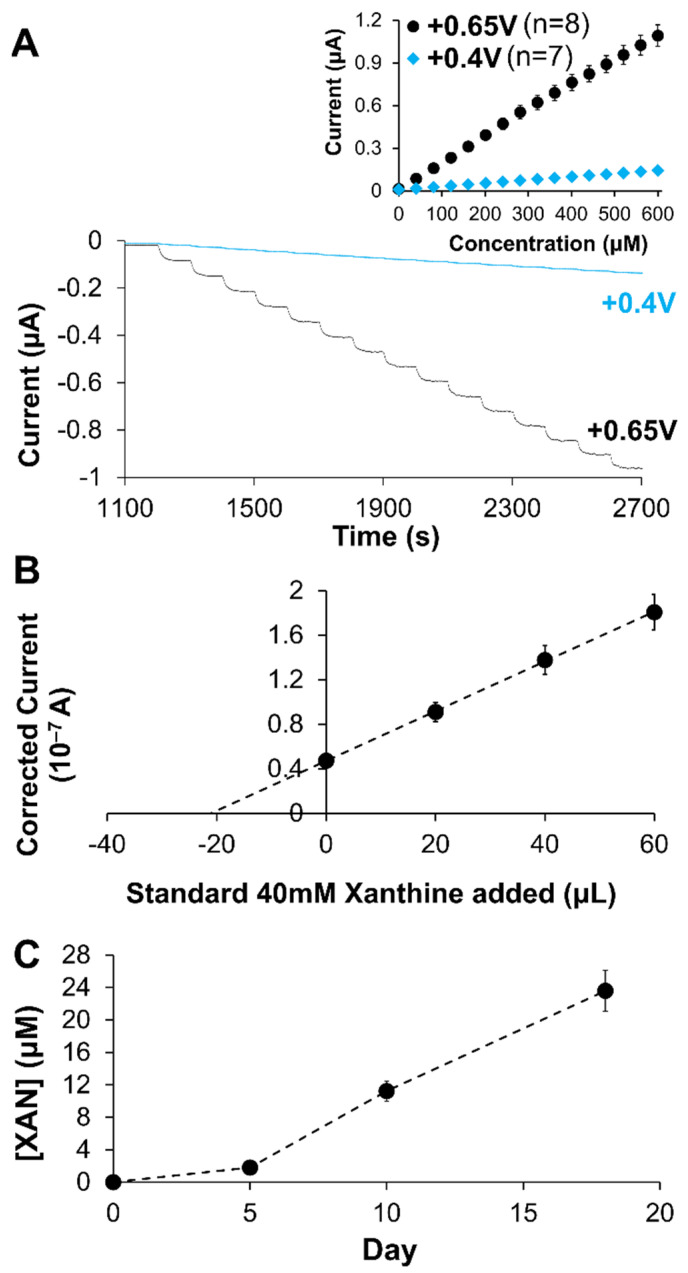
(**A**) Amperometric I-t curves and corresponding calibration curves (inset) for XAN sensing using Pt/HMTES (XOx) + C6-MPCs/PU (75:25) at +0.65 and +0.4 V in synthetic urine samples; (**B**) Typical standard addition plot using Pt/HMTES (XOx) + C6-MPCs/PU (75:25) at +0.65 V in processed fish samples to determine XAN concentration; (**C**) Tracking XAN concentration over time using the biosensor design. Note: Error bars represent standard error; in some cases, error bars are smaller than marker used for average.

The accurate measurement of XAN in urine is important for XAN biosensor if it is to be used as a clinical diagnostic tool for disease detection/monitoring. Similarly, industrial XAN biosensors used for early detection of meat spoilage will require methodologies and functionality compatible with XAN detection in complex media (i.e., food samples) [42]. As such, research and development of XAN biosensors requires evaluating their performance towards these specific applications. In this study, the optimized XAN biosensing schemes of each type of MPC-doped xerogel (i.e., HMTES or TEES) as determined in the studies above were tested for their effectiveness in these applications. For clinical analysis of XAN in a synthetic urine, the modified electrodes were soaked in high ionic strength buffer (65.55 mM; μ = 150 mM; pH 7.0) before a standard XAN calibration curve was collected in a stirred solution of synthetic urine. Examples of the I-t response at both +0.65 V and +0.4 V for the HMTES(XOx) + C6-MPCs system, along with their corresponding calibration curves, are shown in Figure 9A. A similar analysis was also performed using the TEES(XOx) + C6-MPCs system. The results of the calibration curve analysis of synthetic urine spiked with XAN are summarized in the first part of Table 2 and show that when tested at either +0.65 V or +0.4 V, both the HMTES and TEES systems are able to yield percent recoveries of >97% and 70–80%, respectively. It is notable that in this application and using a calibration curve, the HMTES system seems to perform more effectively than the TEES system which was established as a thinner xerogel film and more susceptible to fouling (see below).

A similar testing procedure was followed for calibration curve analysis of XAN in processed fish samples. As seen in the results (Table 2), percent recovery was significantly diminished, in both systems suggesting that the more complex matrix of the processed fish was no longer matrix matched with the calibration curve analysis. Given the known complexity of the fish sample matrix, including significant protein content, standard addition analysis was performed with both the HMTES and TEES systems. Modified electrodes were soaked in PBS, immersed in a fresh (Day 0) fish sample to establish baseline current and then transferred to a processed fish sample spiked with a known XAN concentration or an aged fish sample (i.e., Day X) before being sequentially spiked with three additional standard XAN injections. Figure 9B presents a standard addition plot for this analysis for the HMTES system and the analysis results in ~100% recovery, representing effective sensing of XAN in the fish sample. Notably, the corresponding TEES system exhibited a dampened and disproportional amperometric response once exposed to the fish sample matrix, a likely consequence of electrode fouling. We hypothesize that the more hydrophobic TEES xerogel was likely more susceptible to fouling due to the protein content of the fish samples and denaturation of the proteins at the thinner, hydrophobic interfaces of this particular sensor. Due to this characteristic fouling behavior, the use of the TEES xerogel systems was discontinued. The HMTES+C6-MPCs system was utilized with standard addition analysis on naturally aged fish samples over the course of a few weeks. As shown in Figure 9C, the sensors were able to track increasing XAN over time as the fish meat slowly spoiled, a result consistent with other reports on successful XAN sensors [43,47,51,55,59,70].

## 3. Conclusions

This study systematically examined the use of gold NP doped xerogels as part of LbL modification of electrodes that successfully functioned as first generation, enzymatic xanthine biosensors. The utilization of the gold-NP network within certain xerogel films encapsulating XOx allowed for more a more effective biosensing mechanism resulting in greater sensitivity, expanded linear range, and faster response times. While many of the layers involved require optimization, effective xanthine biosensing was demonstrated for both clinical (urinalysis) and industrial applications (meat spoilage) of xanthine biosensing. The successful design of the xanthine biosensors using nanoparticle-doped xerogels required understanding of applied potential and resulting contributions to signal from the electroactive analytes that may be present during xanthine metabolism over time. Inexpensive and easily operated biosensing platforms that possess both versatility toward applications such as the system presented here, also offering the potential for miniaturization for portable measurements [18], continue to be of significant focus for developing future clinical and industrial sensors targeting analytes of interest.

## 4. Materials and Methods

### 4.1. Materials and Instrumentation

Unless otherwise stated, all chemicals were reagent grade or higher and purchased from Millipore-Sigma (St. Louis, MO, USA). Tecoflex (SG-80A) polyurethane (TPU) and Hydrothane (AL25-80A) polyurethane (HPU) were obtained from Lubrizol (Wickliffe, OH, USA) and AdvanSource Biomaterials (Wilmington, MA, USA), respectively. Hydroxymethyltriethoxysilane (HMTES) was purchased from Gelest Inc. (Morrisville, PA, USA). Electrochemical experiments were conducted using 8-channel potentiostats (CH Instruments, 1000B or 1030C, Bee Cave, TX, USA) or a single channel potentiostat (CH Instruments, 420B) using Ag/AgCl (saturated KCl) aqueous reference electrode (RE) (CH Instruments) and platinum wire counter electrode (CE) (Millipore-Sigma).

### 4.2. Nanoparticle Synthesis

Alkanethiolate-protected gold NPs known as monolayer protected clusters (MPCs) were synthesized using variation of the Brust reaction [39,62]. In brief, a HAuCl_4_ salt solution (aq) was mixed with a toluene solution of tetraoctylammonium bromide, a phase transfer reagent that moves the gold to the toluene layer. To the separated toluene layer, thiol (hexane thiol, butane thiol, or propane thiol) was added in a 2:1 mole ratio with the gold and stirred until a transparent bright orange solution was achieved (~30 min). An ice bath was used to chill both this toluene–thiol mixture as well as a separate mixture of aqueous NaBH_4_ reductant. Upon stirring these two solutions together, a thick, black solid immediately formed in the flask which was subsequently allowed to stir overnight in the ice bath. The organic toluene layer containing the black NPs was separated, rotary evaporated to dryness, precipitated with added acetonitrile, and then vacuum filtered through a medium porosity glass frit (ChemGlass) while washing with additional acetonitrile. As in prior work, this MPC synthesis recipe produced MPC-style NPs of a composition of Au_225_(thiol)_75_ and a core diameter of ~2.0 nm on average as measured via TEM histogram analysis [39].

### 4.3. Preparation of Amperometric Biosensors

Platinum disk working electrodes (CH Instruments) were polished successively with 1.0, 0.3, and 0.05 μm alumina powder and ultrapure (UP) H_2_O (18.02 MΩ·cm) on a polishing wheel. Polished electrodes were subjected to cyclic voltammetry (CV) in 0.1 M H_2_SO_4_ between potentials of +1.2 V and −0.25 at 0.25 V/s until the voltammogram was consistent with that of a clean platinum surface.

Immediately after electrochemical cycling, clean platinum electrodes were first modified with a single xerogel layer (silanes stored in a desiccated glovebox and transferred via 0.5 mL microcentrifuge tubes) as previously reported [18]. Briefly, sol-gel mixtures were prepared by dissolving 4.0, 8.0, or 12.0 mg of xanthine oxidase (XOx) in 75 μL of UP H_2_O in another 0.5 mL microcentrifugation tube. Simultaneously, in a separate vial, 50 μL of the selected silane, with or without ethanol, was diluted with 75 μL of tetrahydrofuran (THF). These tubes were both sealed and mixed by a vortex for about 5 min. After 5 min of individual mixing, 50 μL of the XOx/H_2_O solution was transferred to the tube containing the silane/THF mixture. The new silane/THF and XOx solution was mixed to a consistently uniform sol-gel mixture through flicking and repeated self-pipetting. A similar procedure was followed for MPC-doped xerogels where the MPCs were first added to THF (75 µL) before being vigorously mixed with the silane (1:400 ratio). In either case, a 3 μL aliquot of the final mixture was then deposited directly onto the cleaned platinum electrodes to uniformly cover the entire electrode area. Coated electrodes were immediately placed inside a humidity chamber (50% RH) for 24 h or 48 h to form an aged xerogel that would subsequently be coated with an outer polyurethane (PU) layer (see below). In some cases, as has been previously reported, [18,63] a second un-doped sol-gel layer (i.e., no XOx or AuNPs) was added to the first layer after 8–10 min of aging to form what is considered a second diffusion-limiting xerogel layer in the LbL construction.

The outer PU layer was formed by blending different compositions of hydrophilic (HPU) and hydrophobic (TPU) polymers as described in other studies [18,63]. For example, a 75:25% HPU-TPU blend was prepared by adding 75 mg and 25 mg, respectively, to 5 mL of a THF/ethanol (50/50 *v*/*v*) solution which was subsequently stirred overnight until completely dissolved. The process was facilitated by chopping the polymer beads into smaller pieces and sonicating the mixture. PU blends were replaced with fresh solutions each week. To form the outer-PU layer on the electrode, a 10 μL aliquot was deposited atop the dried (24 h or 48 h) enzyme-bedded xerogels and allowed to dry completely (30 min) before proceeding (see below). Both as-prepared and MPC-doped xerogel films with and without PU capping layers have been characterized in prior work using various microscopies (TEM; SEM; AFM) [18,37,41,63].

### 4.4. Evaluation of Xanthine Biosensors

Sensors were soaked in 10 mM solutions of potassium phosphate buffer (PBS) at pH 7.0 for an hour. After the hour, sensors were stabilized at a potential of +0.65 V for 20 min (1200 s) in 20 mL of 10 mM PBS buffer solution before injecting xanthine. For testing, 20 μL of xanthine (40 mM) was injected into the beaker at 100 s intervals for a total of 15 injections while stirring continuously to obtain a clinically applicable xanthine concentration in the beaker between 40–600 μM. From this amperometric step response of current over time (I-t curve) can be converted to a calibration curve and the data fit for determination of dynamic and linear ranges as well as a measure of sensitivity (slope). Additional sensor parameters (permeability index, selectivity coefficient, and response time) were measured as demonstrated in previous reports [R63] with details provided in Supporting Information.

### 4.5. Evaluation of Xanthine Biosensors in Simulated Clinical Samples (Urine)

Evaluation of optimized XAN biosensors for clinical and industrial applications focused on the detection of XAN in a different sample matrices. For clinical applications, optimized biosensor assemblies were constructed and soaked for 1 h in high ionic strength PBS (65.55 mM; μ = 150 mM; pH 7.0) for matrix matching the ionic strength of a synthetic urine solution (Sigmatrix Urine Diluent, Sigma-Aldrich, St. Louis, MO, USA). After soaking, the sensors were quickly transferred to the stirred synthetic urine media and the amperometry experiment was initiated, allowing for 20 min of equilibration in stirred synthetic urine prior to the first standard XAN injection. During subsequent successive XAN standard injections, a stair-step current response was collected and the resulting I-t responses translated to a linear calibration curve. After generating the calibration curve in synthetic urine, the soaking procedure was repeated on the same sensors before inserting them into a new synthetic urine solution, allowing for equilibration, and then subsequently spiked with a known amount of XAN to determine percent recovery.

### 4.6. Evaluation of Xanthine Biosensors in Real Samples (Fish Meat)

Fish processing and testing was based on variations of procedures previously reported in the literature [47,55,56]. Fresh rock fish (sea bass), caught on 15 March 2023 and rendered on 19 March 2023 was purchased at the market on 19 March 2023. The fish meat was portioned into 3 g segments and either frozen (Day 0), refrigerated, or set-aside in the open air (fume hood) for aging over days. Each fish portion was homogenized in a food processor (Magic Bullet Blender) with 10 mL of chilled PBS (65.55 mM; μ = 150 mM; pH 7.0) for at least 5 min until a white, frothy solution was achieved. This solution was then filtered using slight vacuum through a Buchner funnel lined with muslin cloth (Joann Fabrics) [51,55]. An additional 5–10 mL of the chilled PBS was used to rinse the blender and added to the filtration. The filtrate was then quantitatively transferred (Pasteur pipet and minimal additional PBS) to a centrifuge tube (50 mL; Oak Ridge) and the sample was centrifuged (Beckman: 13,000× *g* rpm; 4 °C for 20 min). The supernatant liquid was carefully transferred to a 25 mL volumetric flask and diluted to volume with PBS (65.55 mM; μ = 150 mM; pH 7.0).

For calibration curve analysis of processed fish samples, modified electrodes were first soaked in PBS (65.55 mM; μ = 150 mM; pH 7.0) before a standardized I-t curve during XAN injections was collected (stirred solution). The same electrodes were then rinsed, immersed in a stirred, fresh (i.e., Day 0) sample of processed fish solution while under potential control in order to establish non-Faradaic charging current baseline in that media (~200 s). While still under potential control, the electrodes were then quickly transferred to a XAN spiked solution of stirred process fish sample and the amperometric response recorded.

For each standard addition analysis, two fish sample solutions were prepared as described above in tandem: a Day 0 sample for a baseline measurement and a sample that had been aged for a specific number of days (Day X) or spiked with standard XAN. The modified electrodes were initially soaked for 1 h in PBS (65.55 mM; μ = 150 mM; pH 7.0) before an I-t curve in PBS was collected over the course of ~900 s. At that point, the electrodes still under potential control, were quickly transferred to a stirred solution of 20 mL of Day 0 fish sample so as to establish a stable baseline (~200–300 s) of charging current in the complex sample matrix with minimal XAN present). After baseline stabilization, the electrodes are again transferred to a stirred fish sample (i.e., spiked with XAN or aged for a number of days) and the signal was allowed to stabilize again. To this stirred solution, three standard XAN injections (20 μL of 40.32 mM XAN) were then made at ~100 s apart, creating a stair-step response for a standard addition plot.

## Figures and Tables

**Table 1:** Sensitivity, Calibration Curve Linearity, and Response Times of XAN Biosensors with Pt Electrodes Modified with HMTES or TEES Xerogels and Capped with Polyurethane.

LbL Assembly Scheme at Pt Electrode	*n*	Sensitivity ^a^ (nA/μM)	Correction Coefficient ^b^ (R^2^)	Response Time, ^c^ t_r-95%_ (s)
**HMTES**				
HMTES (XOx)/PU	5	1.02	0.9926	39.3 (±2.8)
HMTES (XOx) + C3-MPCs/PU	5	0.95	0.9663	38.7 (±1.3)
HMTES (XOx) + C4-MPCs/PU	5	1.86	0.9997	31.3 (±0.8)
HMTES (XOx) + C6-MPCs/PU *	**10**	**2.34**	**0.9995**	**19.1 (±1.5)**
HMTES (XOx)/HMTES/PU	6	0.40	0.9995	42.0 (±2.4)
HMTES (XOx) + C3-MPCs/HMTES/PU	4	0.47	0.9761	26.8 (± 2.7)
HMTES (XOx) + C4-MPCs/HMTES/PU	4	0.76	0.9960	23.0 (±1.1)
HMTES (XOx) + C6-MPCs/HMTES/PU	3	0.99	0.9997	22.4 (±1.9)
**TEES**				
TEES (XOx)/PU	5	2.06	0.9895	59.2 (±4.1)
TEES (XOx) + C3-MPCs/PU	4	2.70	0.9856	27.2 (±9.3)
TEES (XOx) + C4-MPCs/PU *	**4**	**3.08**	**0.9915**	**19.4 (±2.9)**
TEES (XOx) + C6-MPCs/PU	3	2.67	0.9894	55.5 (±2.1)
TEES (XOx)/TEES/PU	6	*No signal*	*---*	*---*
TEES (XOx) + C3-MPCs/TEES/PU	-	*---*	*---*	*---*
TEES (XOx) + C4-MPCs/TEES/PU	-	*---*	*---*	*---*
TEES (XOx) + C6-MPCs/TEES/PU	-	*---*	*---*	*---*

**Notes:** All systems modified a platinum electrode and were capped with PU blend ratio of 75:25 HPU:TPU (*w*/*w*). ^a^ Relative standard deviation ranged from 5–10% on all systems. ^b^ In all cases the use of MPCs improved the linear fit and extended the linear range from 0–280 μM to 0–600 μM (HMTES system) and from 0–240 μM to 0–480 μM (TEES system). ^c^ Response time determined as time for signal to reach 95% of steady-state response.

**Table 2:** Xanthine Biosensing Performance in Clinical (Urine Analysis) and Industrial (Fish Spoilage) Applications.

LbL Assembly Scheme at Pt Electrode	E_app_ (V)	Method	*n*	(XAN) Spike (μM)	(XAN) Found (μM)	% Recovery
**Synthetic Urine**						
HMTES (XOx) + C6-MPCs/PU	+0.65	CC	8	80.5	78.3_(±1.7)_	97.2_(±2.1)_
HMTES (XOx) + C6-MPCs/PU	+0.4	CC	7	80.5	79.3_(±2.1)_	98.5_(±2.6)_
TEES (XOx) + C6-MPCs/PU	+0.65	CC	4	300.1	233.6_(±26.7)_	77.8_(±8.9)_
TEES (XOx) + C6-MPCs/PU	+0.4	CC	4	300.1	213.9_(±28.7)_	71.3_(±9.6)_
TEES (XOx) + C4-MPCs/PU	+0.65	CC	4	80.5	57.8_(±9.6)_	71.8_(±7.9)_
TEES (XOx) + C4-MPCs/PU	+0.4	CC	3	80.5	49.2_(±3.8)_	61.1_(±4.7)_
**Sea Bass**						
HMTES (XOx) + C6-MPCs/PU	+0.65	CC	7	42.0	23.6_(±2.1)_	59.5_(±5.0)_
HMTES (XOx) + C6-MPCs/PU	+0.65	SA	6	40.3	42.2_(±1.1)_	104.8_(±2.6)_
TEES (XOx) + C6-MPCs/PU	+0.65	CC	4	42.0	19.2_(±3.6)_	45.7_(±8.7)_
TEES (XOx) + C6-MPCs/PU	+0.65	SA	*Fouled*			

Notes: All systems modified a platinum electrode and were capped with PU blend ratio of 75:25 HPU:TPU (*w*/*w*); Uncertainty values reflect standard error.

## Data Availability

The data presented in this study are available on request from the corresponding author.

## Supplementary Materials

The following supporting information can be downloaded at: https://www.mdpi.com/article/10.3390/gels9060437/s1, Scheme S1: Mechanistic pathway of purine metabolism; Figure S1: Cyclic voltammetry of potassium ferricyanide at one and two layers of HMTES or TEES xerogel layers; Figure S2: Summary of permeability indices for peroxide and xanthine measured at various formulations of different sol-gel mixtures to form different xerogels; Figure S3: Xanthine calibration curves for the Pt/TEES (XOx)/PU (75:25) system with various loadings of XOx enzyme in the xerogel deposition mixtures (i.e., enzyme loading effects); Scheme S2: Illustration of NP-doped xerogel signal enhancement; Figure S4–S6: Xanthine calibration curves for MPC-doped and undoped xerogel layers for both the HMTES and TEES systems; Figure S7: Xanthine calibration curves of MPC-doped TEES systems monitored over a week for sensitivity (slope); Figure S8: Xanthine and hypoxanthine calibration curves for Pt/TEES (XOx) + C6 MPC/PU (75:25) system; Figure S9–S11: Selectivity coefficient measurements over time for common interferents at MPC-doped systems of both HMTES and TEES sensors; Figure S12–S14: Cyclic voltammetry of 100 mM solutions of xanthine, uric acid, and hypoxanthine (in PBS) at a Pt electrode; Figure S15–S16: Amperometric I-t curves and summary result table from UA and H_2_O_2_ injections at Pt electrode held at various potentials; Table S1: Current response and response ratios of H_2_O_2_/Uric Acid at bare Pt electrode held at different potentials.

## Abbreviations

AFM—atomic force microscopy; Au-NPs—gold nanoparticles; CAT—catalase; CE—counter electrode; CNT—carbon nanotubes; CV—cyclic voltammetry; FeCN—potassium ferricyanide; HMTES—hydroxymethyl triethoxy silane; HPU—Hydrothane polyurethane; HXAN—hypoxanthine; LBL—layer-by-layer; LOD—limit of detection; MPC—monolayer protected clusters; NMs—nanomaterials; NP—nanoparticles; PBS—potassium phosphate buffer; PI—permeability index; POC—point of care; PTMS—propyltrimethoxy silane; PU—polyurethane layer; RE—reference electrode; ROS—reactive oxygen species; SEM—scanning electron microscopy; TEES—triethoxyethyl silane; TEM—transmission electron microscopy; TPU—Tecoflex polyurethane; UA—uric acid; UP—ultra pure water (18.02 MΩ·cm); XAN—xanthine; XOx—xanthine oxidase.
