# Supplementary material for: Nanomaterial-Doped Xerogels for Biosensing Measurements of Xanthine in Clinical and Industrial Applications
Source: Gels. 2023 May 25;9(6):437. doi: 10.3390/gels9060437 (PMC10297527; doi:10.3390/gels9060437)
Supplement: Supplementary file 1 [file gels-09-00437-s001.zip › gels-2414705-supplementary.pptx]

## Slide 1
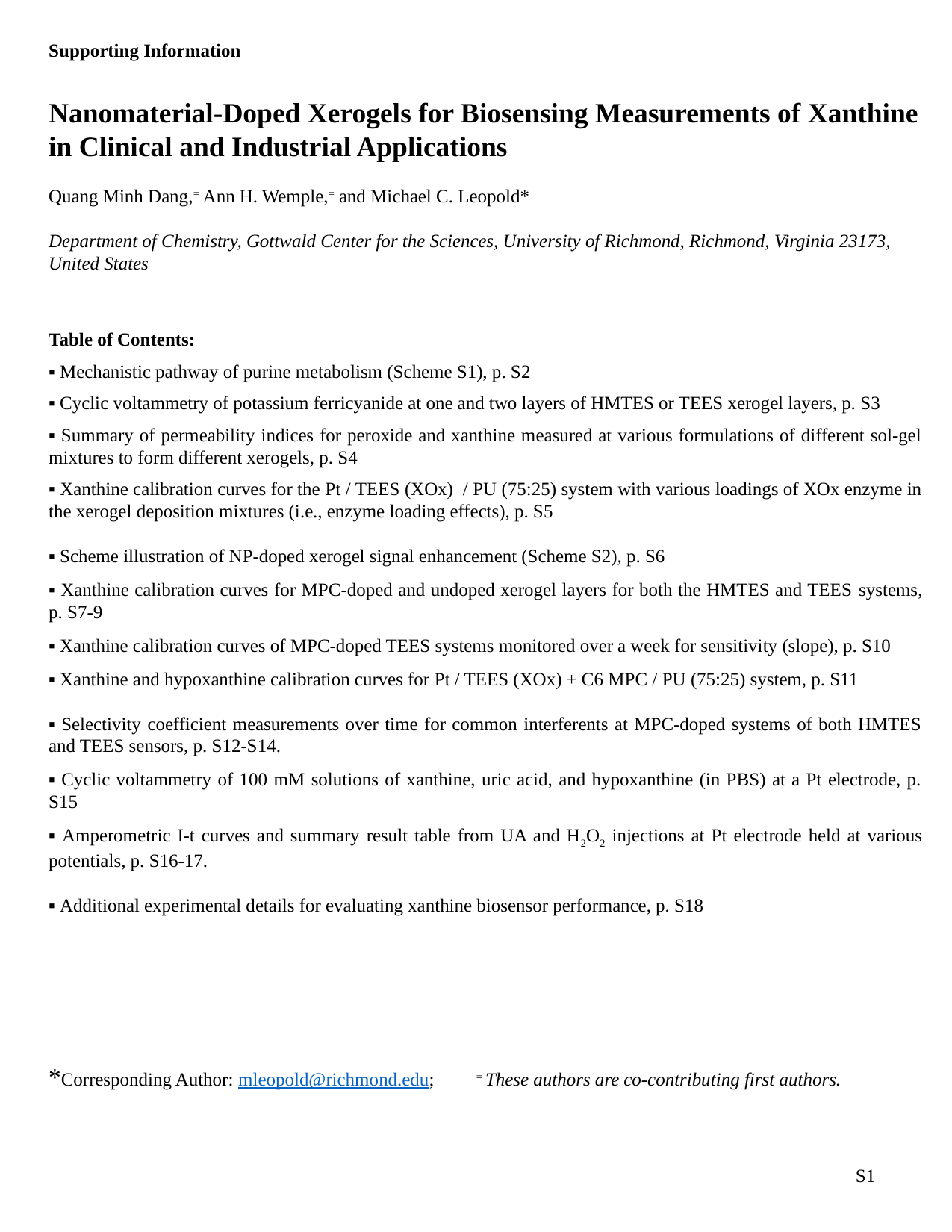

Supporting Information
Nanomaterial-Doped Xerogels for Biosensing Measurements of Xanthine in Clinical and Industrial Applications
Quang Minh Dang,= Ann H. Wemple,= and Michael C. Leopold*
Department of Chemistry, Gottwald Center for the Sciences, University of Richmond, Richmond, Virginia 23173, United States
Table of Contents:
▪ Mechanistic pathway of purine metabolism (Scheme S1), p. S2
▪ Cyclic voltammetry of potassium ferricyanide at one and two layers of HMTES or TEES xerogel layers, p. S3
▪ Summary of permeability indices for peroxide and xanthine measured at various formulations of different sol-gel mixtures to form different xerogels, p. S4
▪ Xanthine calibration curves for the Pt / TEES (XOx) / PU (75:25) system with various loadings of XOx enzyme in the xerogel deposition mixtures (i.e., enzyme loading effects), p. S5
▪ Scheme illustration of NP-doped xerogel signal enhancement (Scheme S2), p. S6
▪ Xanthine calibration curves for MPC-doped and undoped xerogel layers for both the HMTES and TEES systems, p. S7-9
▪ Xanthine calibration curves of MPC-doped TEES systems monitored over a week for sensitivity (slope), p. S10
▪ Xanthine and hypoxanthine calibration curves for Pt / TEES (XOx) + C6 MPC / PU (75:25) system, p. S11
▪ Selectivity coefficient measurements over time for common interferents at MPC-doped systems of both HMTES and TEES sensors, p. S12-S14.
▪ Cyclic voltammetry of 100 mM solutions of xanthine, uric acid, and hypoxanthine (in PBS) at a Pt electrode, p. S15
▪ Amperometric I-t curves and summary result table from UA and H2O2 injections at Pt electrode held at various potentials, p. S16-17.
▪ Additional experimental details for evaluating xanthine biosensor performance, p. S18
*Corresponding Author: mleopold@richmond.edu; = These authors are co-contributing first authors.
S1

## Slide 2
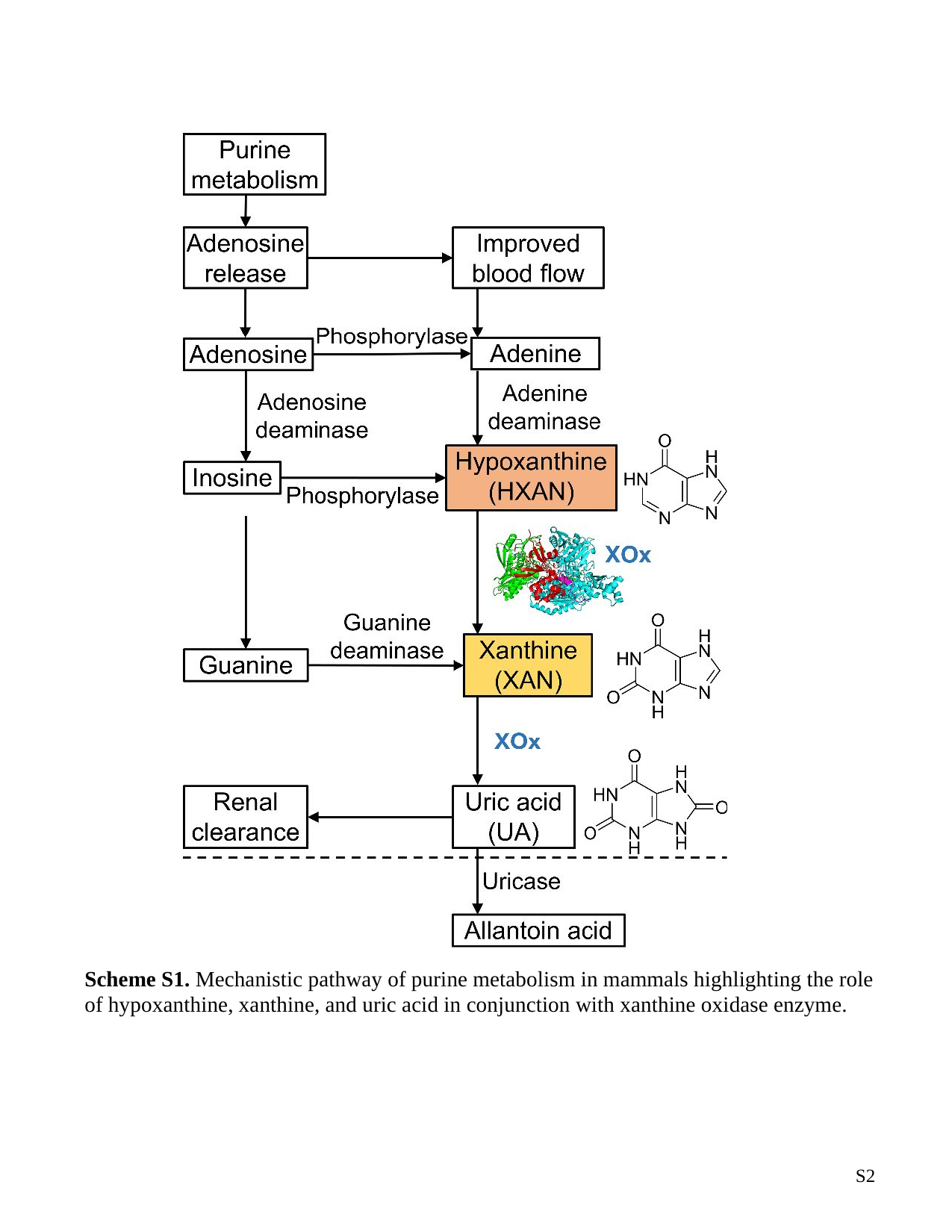

Scheme S1. Mechanistic pathway of purine metabolism in mammals highlighting the role of hypoxanthine, xanthine, and uric acid in conjunction with xanthine oxidase enzyme.
S2

## Slide 3
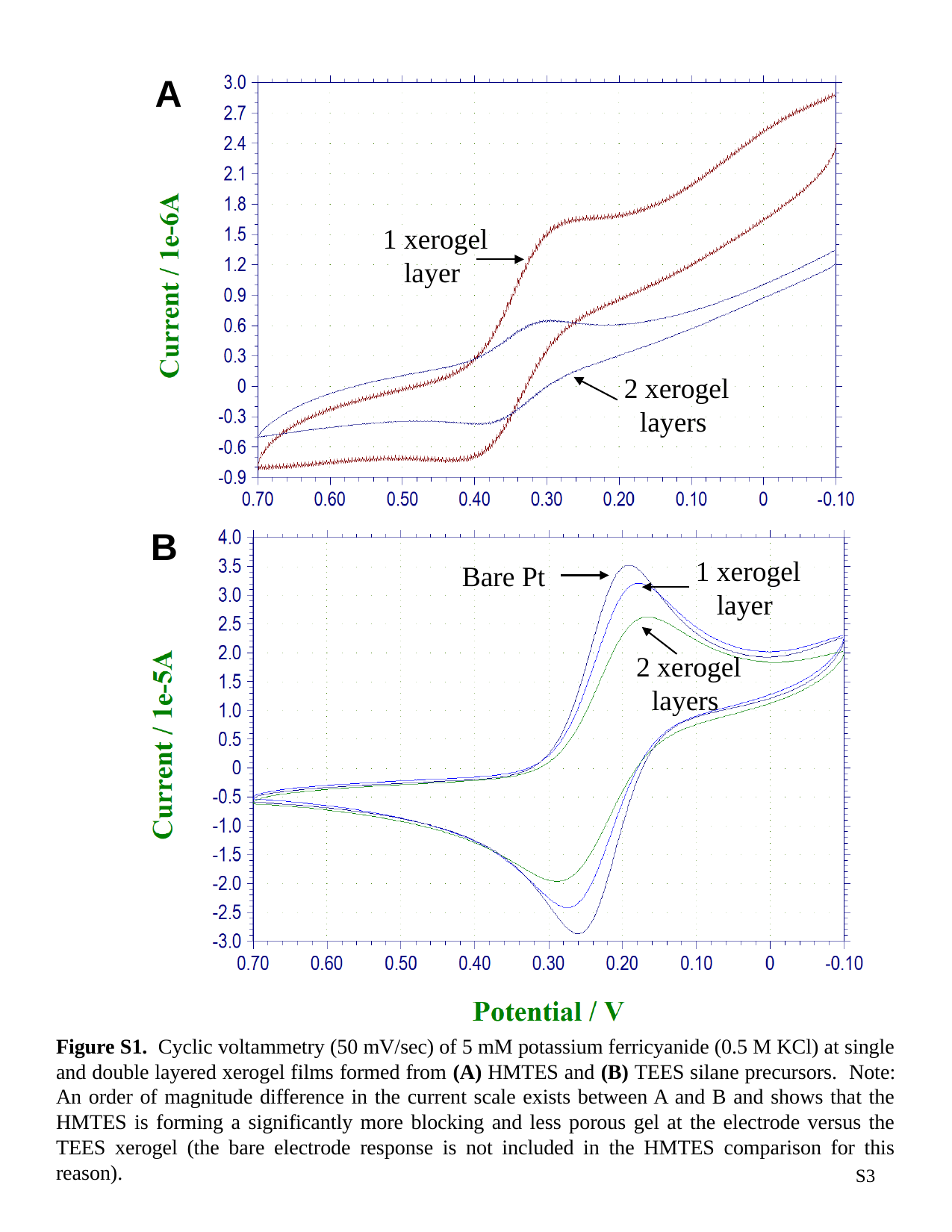

A
1 xerogel
layer
2 xerogel
layers
B
1 xerogel
layer
Bare Pt
2 xerogel
layers
Figure S1. Cyclic voltammetry (50 mV/sec) of 5 mM potassium ferricyanide (0.5 M KCl) at single and double layered xerogel films formed from (A) HMTES and (B) TEES silane precursors. Note: An order of magnitude difference in the current scale exists between A and B and shows that the HMTES is forming a significantly more blocking and less porous gel at the electrode versus the TEES xerogel (the bare electrode response is not included in the HMTES comparison for this reason).
S3

## Slide 4
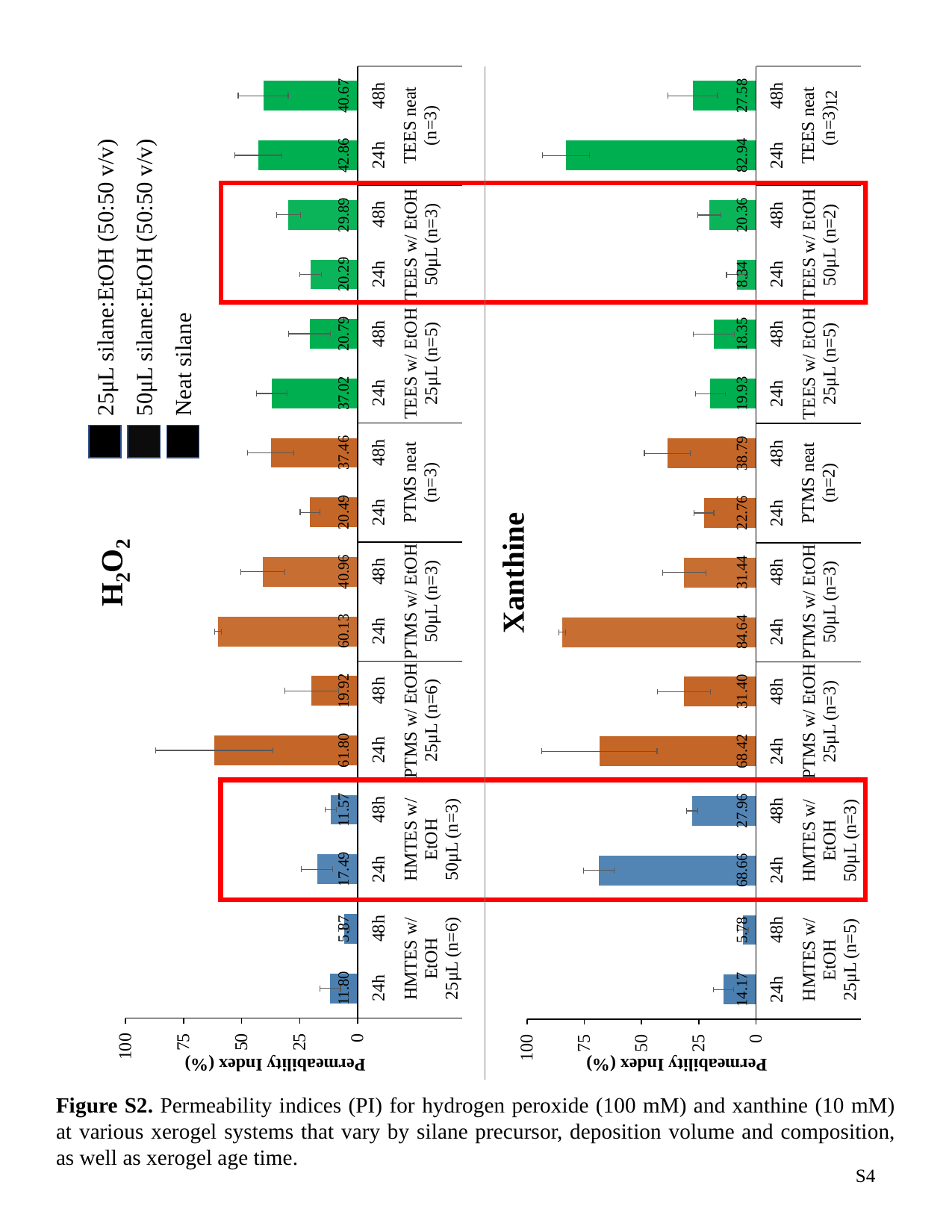

Figure S2. Permeability indices (PI) for hydrogen peroxide (100 mM) and xanthine (10 mM) at various xerogel systems that vary by silane precursor, deposition volume and composition, as well as xerogel age time.
S4

## Slide 5
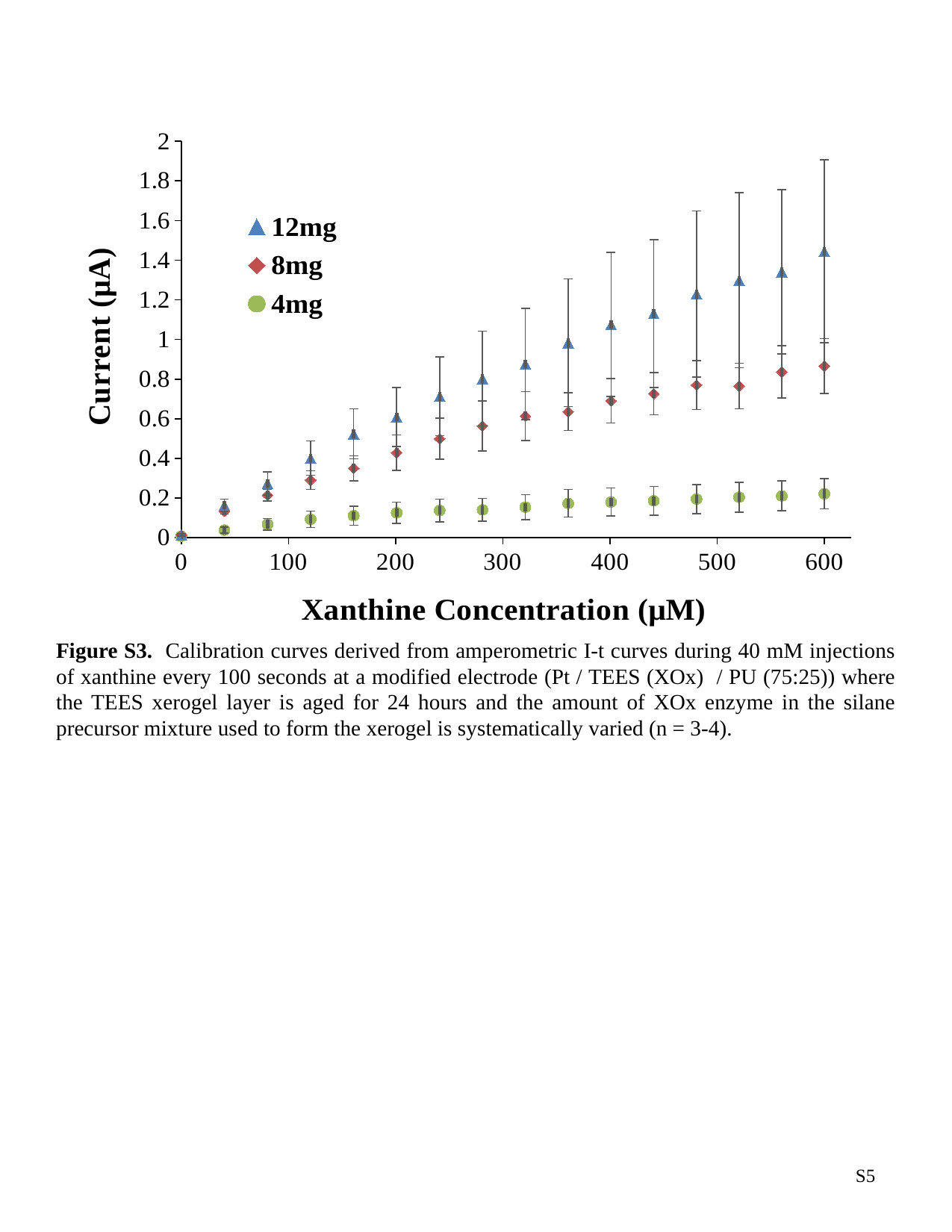

### Chart
| Category | 12mg | 8mg | 4mg |
|---|---|---|---|Figure S3. Calibration curves derived from amperometric I-t curves during 40 mM injections of xanthine every 100 seconds at a modified electrode (Pt / TEES (XOx) / PU (75:25)) where the TEES xerogel layer is aged for 24 hours and the amount of XOx enzyme in the silane precursor mixture used to form the xerogel is systematically varied (n = 3-4).
S5

## Slide 6
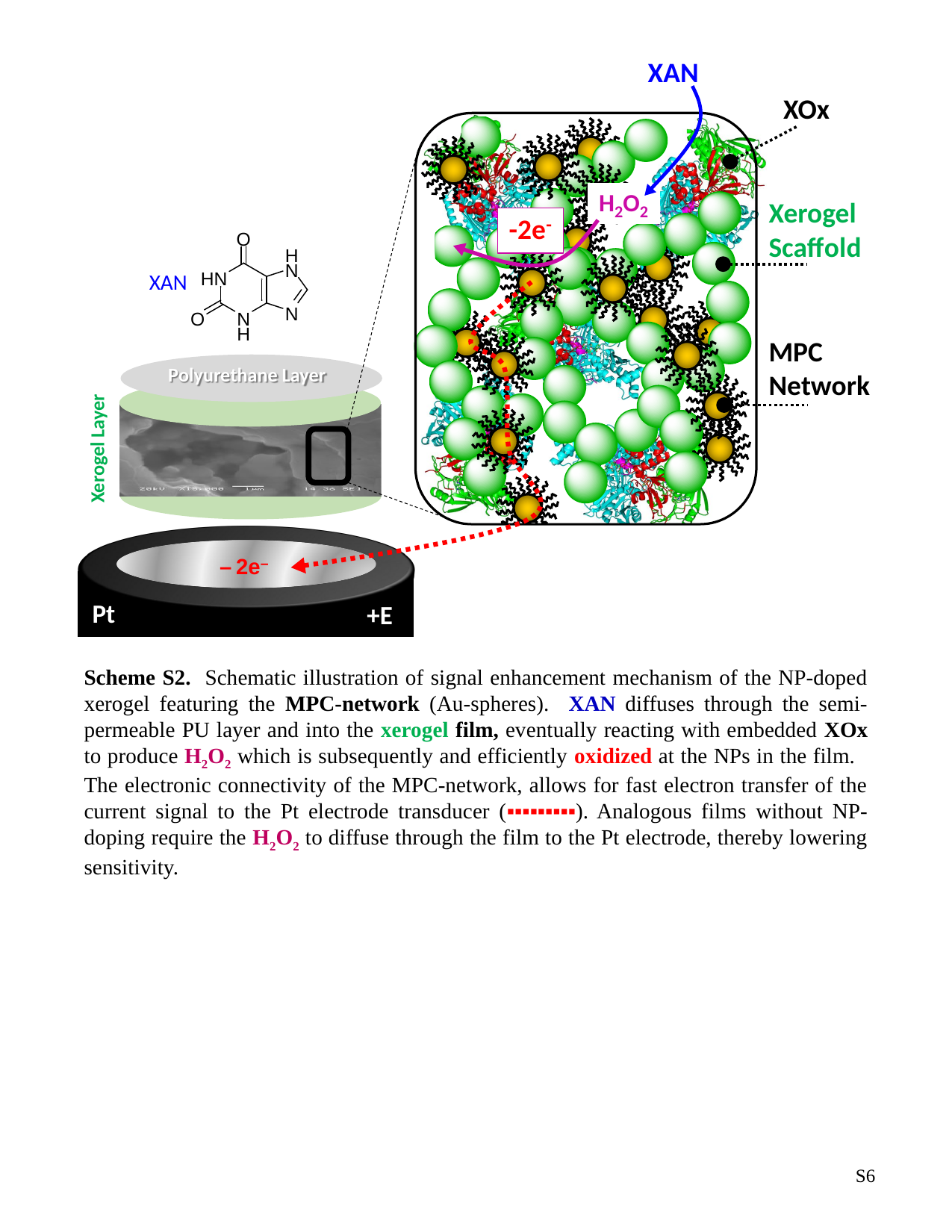

Scheme S2. Schematic illustration of signal enhancement mechanism of the NP-doped xerogel featuring the MPC-network (Au-spheres). XAN diffuses through the semi-permeable PU layer and into the xerogel film, eventually reacting with embedded XOx to produce H2O2 which is subsequently and efficiently oxidized at the NPs in the film. The electronic connectivity of the MPC-network, allows for fast electron transfer of the current signal to the Pt electrode transducer (▪▪▪▪▪▪▪▪▪). Analogous films without NP-doping require the H2O2 to diffuse through the film to the Pt electrode, thereby lowering sensitivity.
S6

## Slide 7
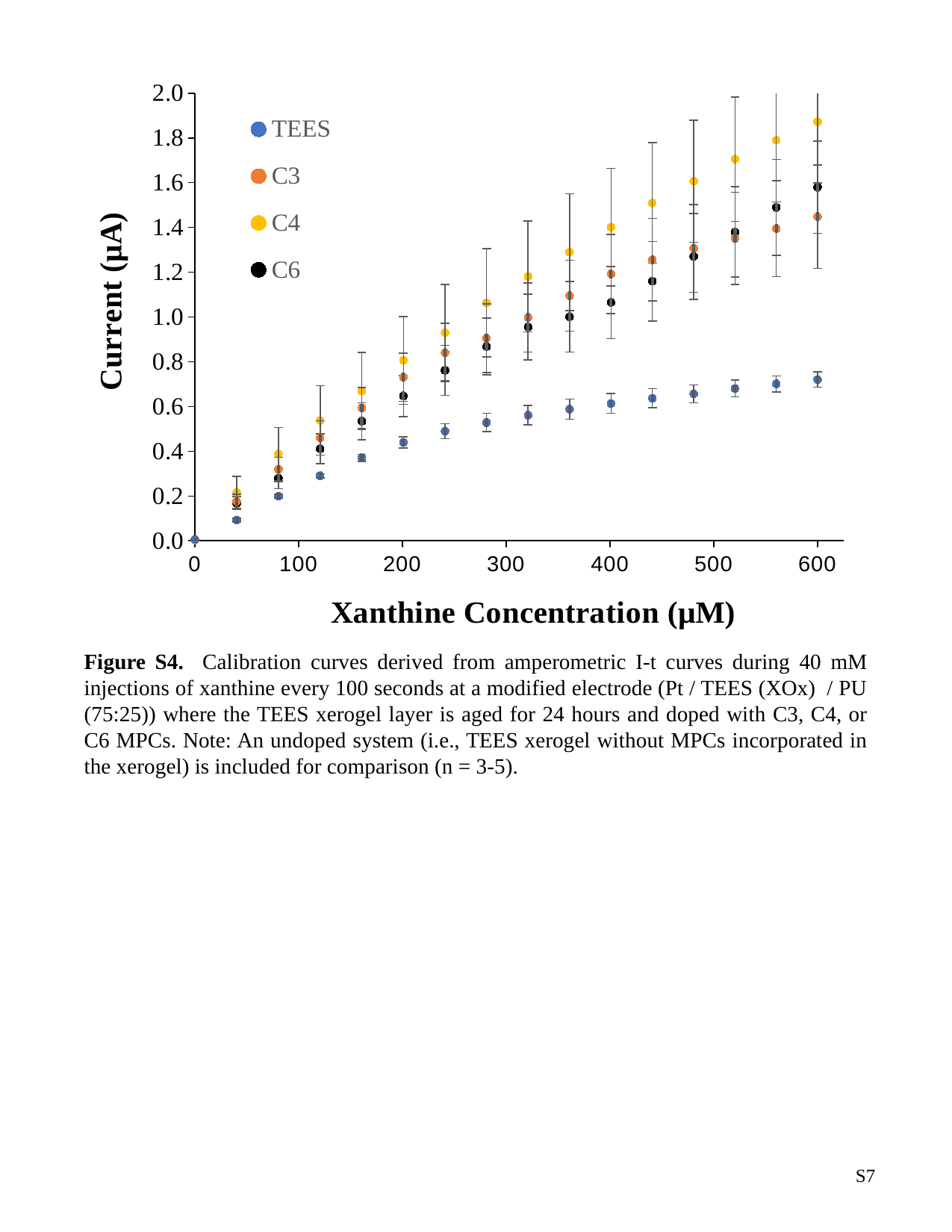

### Chart
| Category | TEES | C3 | | C6 |
|---|---|---|---|---|Figure S4. Calibration curves derived from amperometric I-t curves during 40 mM injections of xanthine every 100 seconds at a modified electrode (Pt / TEES (XOx) / PU (75:25)) where the TEES xerogel layer is aged for 24 hours and doped with C3, C4, or C6 MPCs. Note: An undoped system (i.e., TEES xerogel without MPCs incorporated in the xerogel) is included for comparison (n = 3-5).
S7

## Slide 8
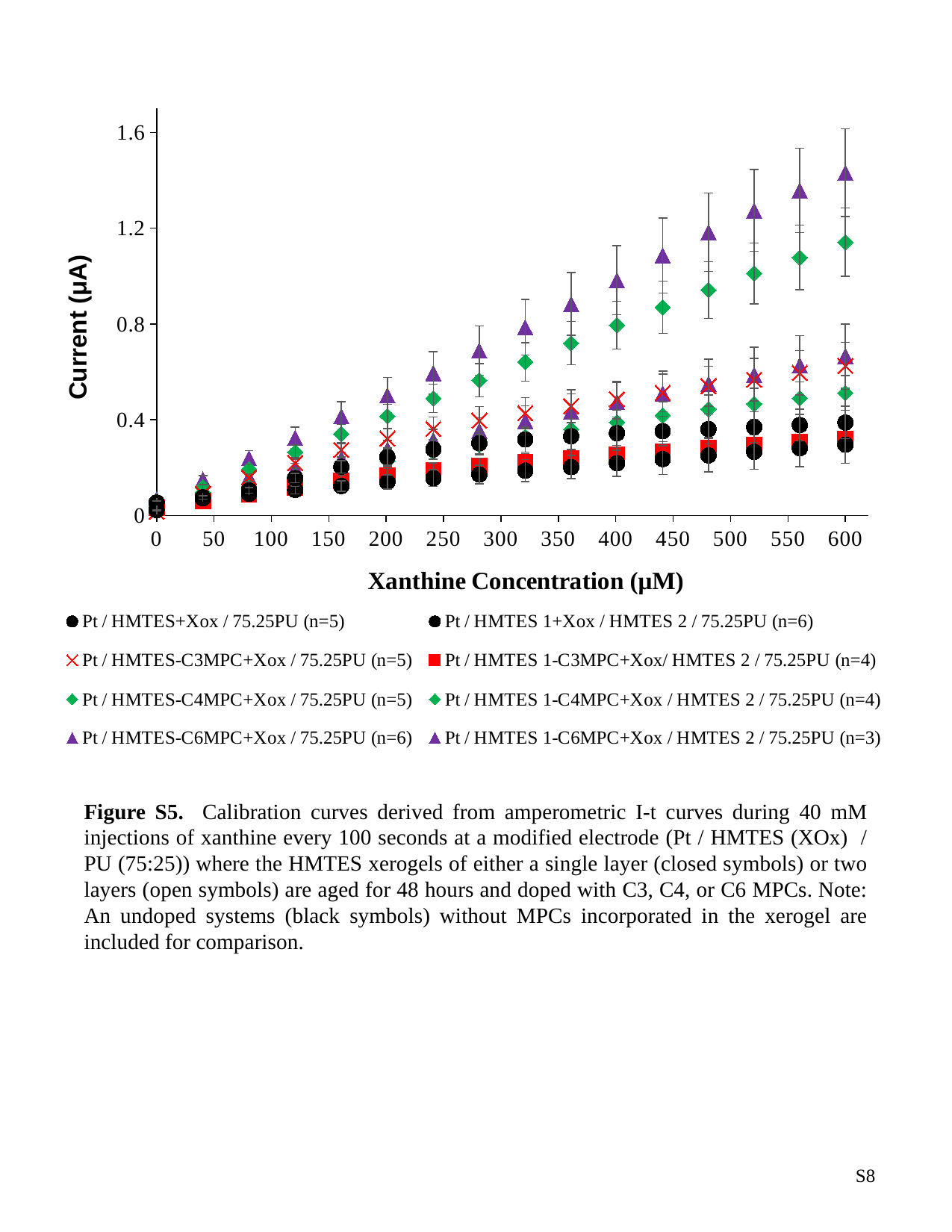

### Chart
| Category | Pt / HMTES+Xox / 75.25PU (n=5) | Pt / HMTES 1+Xox / HMTES 2 / 75.25PU (n=6) | Pt / HMTES-C3MPC+Xox / 75.25PU (n=5) | Pt / HMTES 1-C3MPC+Xox/ HMTES 2 / 75.25PU (n=4) | Pt / HMTES-C4MPC+Xox / 75.25PU (n=5) | Pt / HMTES 1-C4MPC+Xox / HMTES 2 / 75.25PU (n=4) | Pt / HMTES-C6MPC+Xox / 75.25PU (n=6) | Pt / HMTES 1-C6MPC+Xox / HMTES 2 / 75.25PU (n=3) |
|---|---|---|---|---|---|---|---|---|Figure S5. Calibration curves derived from amperometric I-t curves during 40 mM injections of xanthine every 100 seconds at a modified electrode (Pt / HMTES (XOx) / PU (75:25)) where the HMTES xerogels of either a single layer (closed symbols) or two layers (open symbols) are aged for 48 hours and doped with C3, C4, or C6 MPCs. Note: An undoped systems (black symbols) without MPCs incorporated in the xerogel are included for comparison.
8
S8

## Slide 9
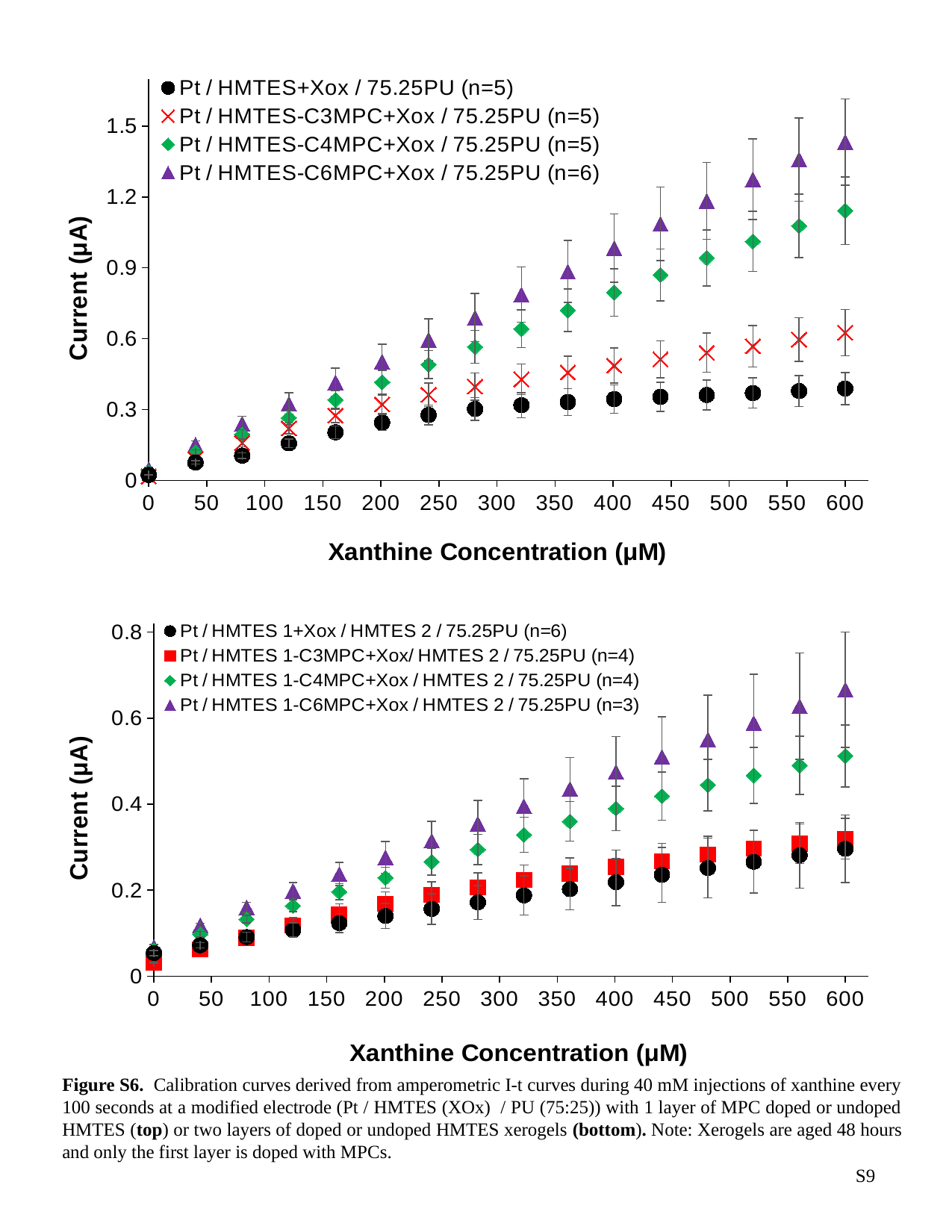

### Chart
| Category | Pt / HMTES+Xox / 75.25PU (n=5) | Pt / HMTES-C3MPC+Xox / 75.25PU (n=5) | Pt / HMTES-C4MPC+Xox / 75.25PU (n=5) | Pt / HMTES-C6MPC+Xox / 75.25PU (n=6) |
|---|---|---|---|---|
### Chart
| Category | Pt / HMTES 1+Xox / HMTES 2 / 75.25PU (n=6) | Pt / HMTES 1-C3MPC+Xox/ HMTES 2 / 75.25PU (n=4) | Pt / HMTES 1-C4MPC+Xox / HMTES 2 / 75.25PU (n=4) | Pt / HMTES 1-C6MPC+Xox / HMTES 2 / 75.25PU (n=3) |
|---|---|---|---|---|Figure S6. Calibration curves derived from amperometric I-t curves during 40 mM injections of xanthine every 100 seconds at a modified electrode (Pt / HMTES (XOx) / PU (75:25)) with 1 layer of MPC doped or undoped HMTES (top) or two layers of doped or undoped HMTES xerogels (bottom). Note: Xerogels are aged 48 hours and only the first layer is doped with MPCs.
S9

## Slide 10
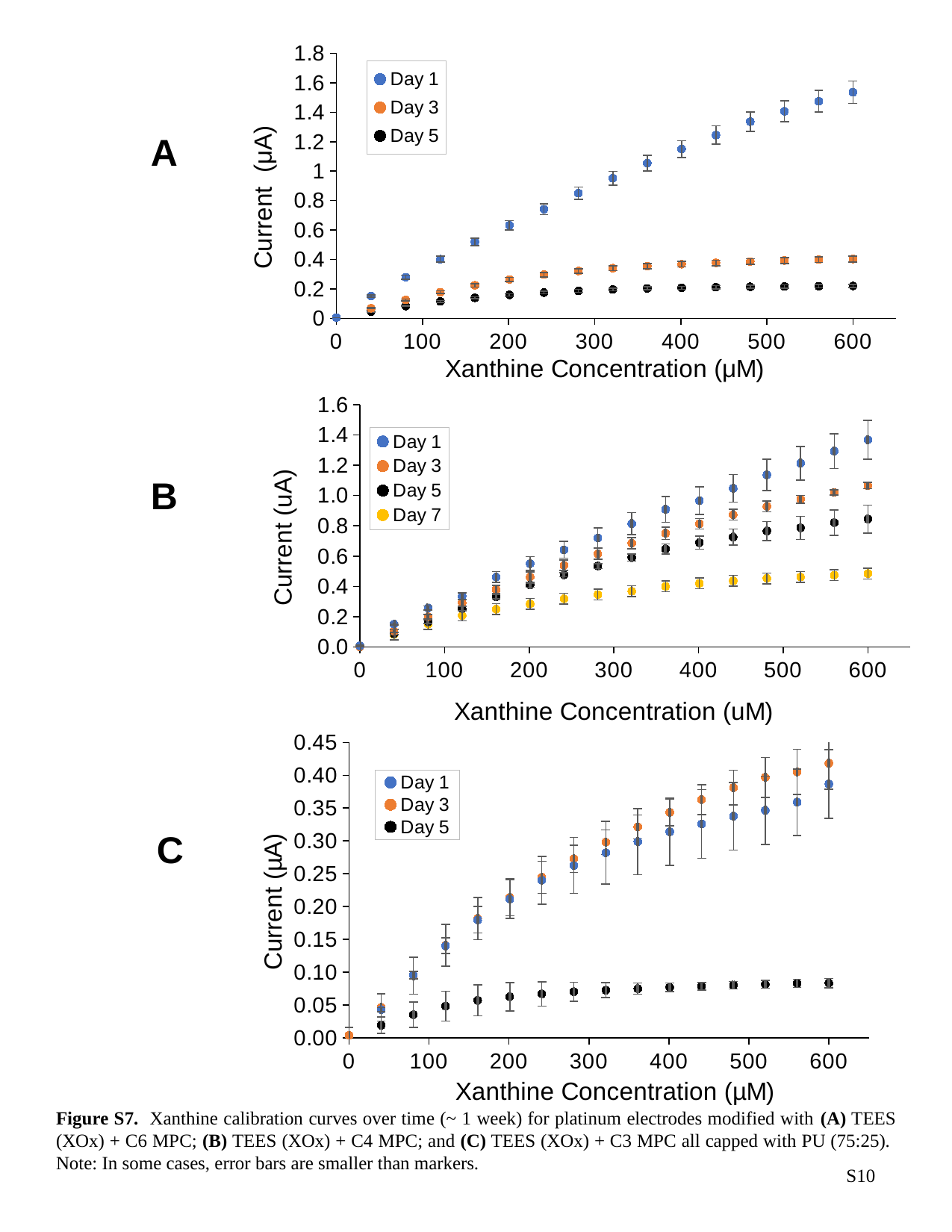

### Chart
| Category | | | |
|---|---|---|---|A
### Chart
| Category | | | | |
|---|---|---|---|---|B
### Chart
| Category | Day 1 | Day 3 | Day 5 |
|---|---|---|---|C
Figure S7. Xanthine calibration curves over time (~ 1 week) for platinum electrodes modified with (A) TEES (XOx) + C6 MPC; (B) TEES (XOx) + C4 MPC; and (C) TEES (XOx) + C3 MPC all capped with PU (75:25). Note: In some cases, error bars are smaller than markers.
S10

## Slide 11
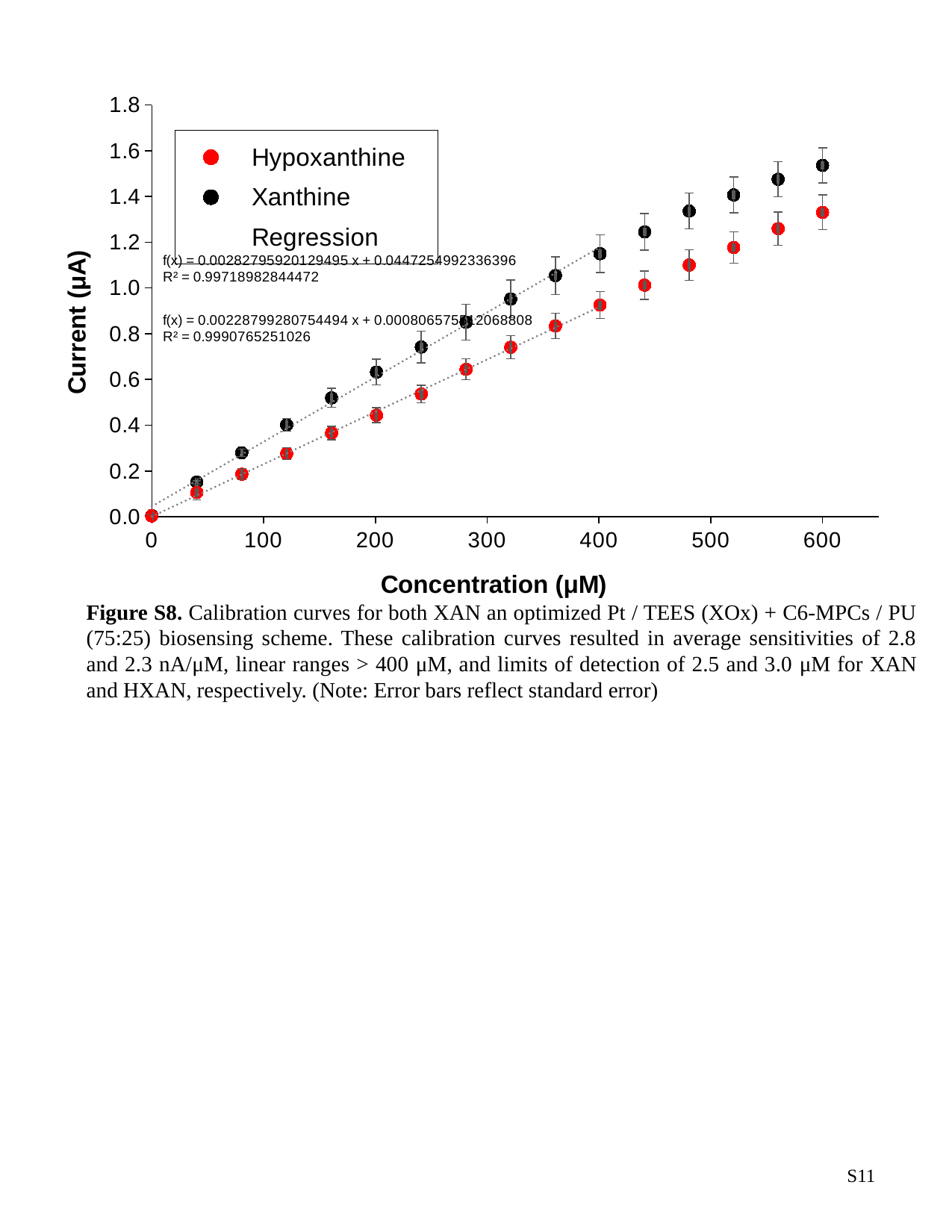

### Chart
| Category | Hypoxanthine | Xanthine | | |
|---|---|---|---|---|Figure S8. Calibration curves for both XAN an optimized Pt / TEES (XOx) + C6-MPCs / PU (75:25) biosensing scheme. These calibration curves resulted in average sensitivities of 2.8 and 2.3 nA/μM, linear ranges > 400 μM, and limits of detection of 2.5 and 3.0 μM for XAN and HXAN, respectively. (Note: Error bars reflect standard error)
S11

## Slide 12
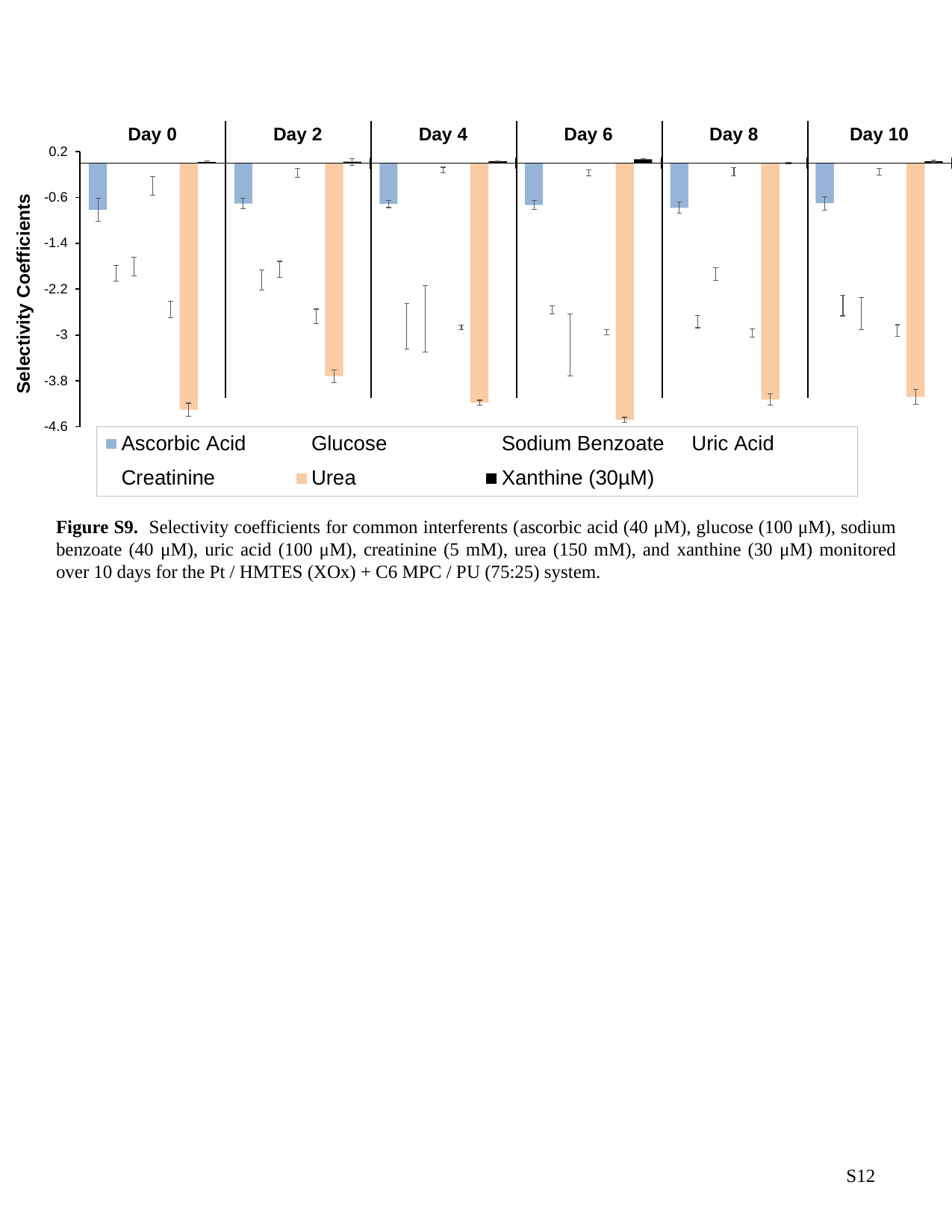

Figure S9. Selectivity coefficients for common interferents (ascorbic acid (40 μM), glucose (100 μM), sodium benzoate (40 μM), uric acid (100 μM), creatinine (5 mM), urea (150 mM), and xanthine (30 μM) monitored over 10 days for the Pt / HMTES (XOx) + C6 MPC / PU (75:25) system.
S12

## Slide 13
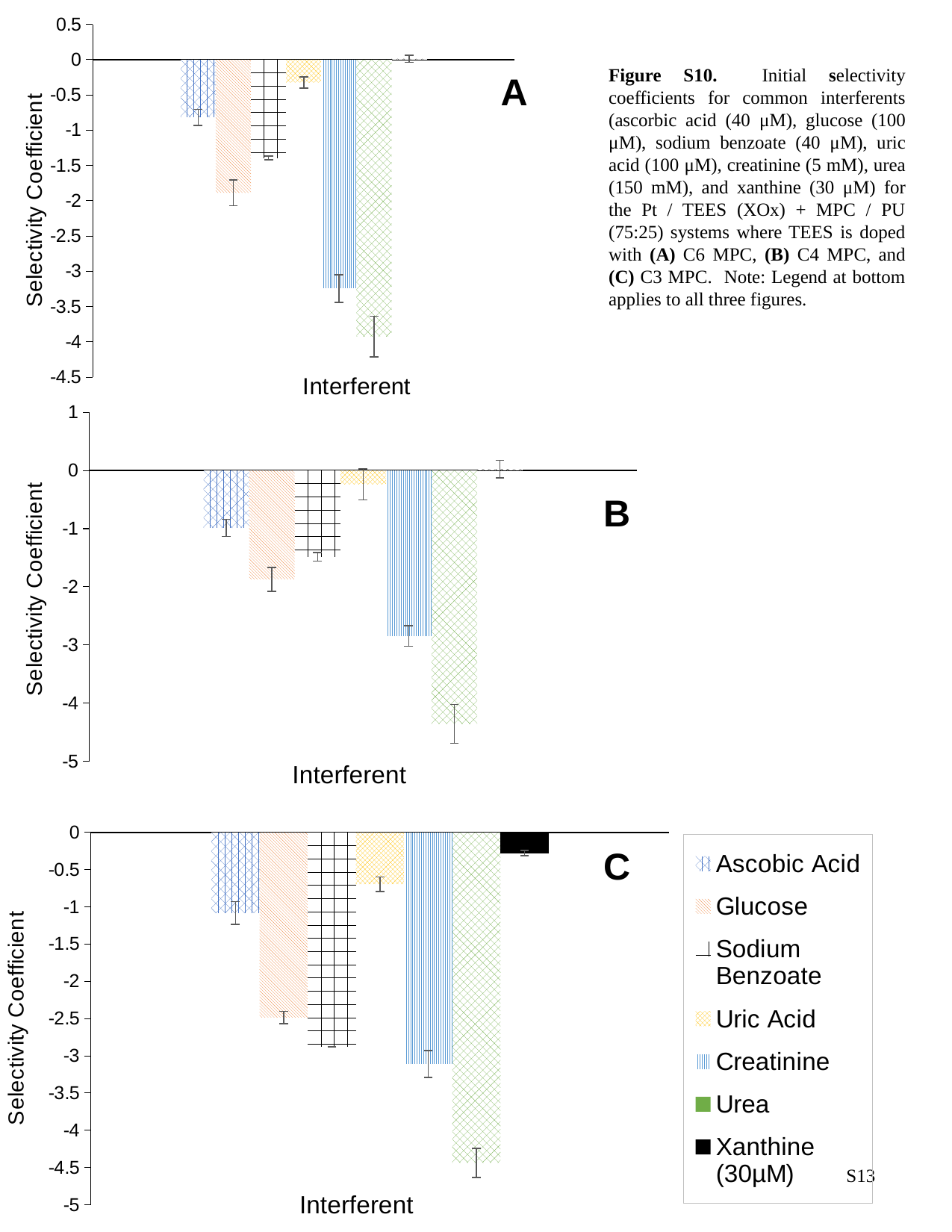

### Chart
| Category | Ascobic Acid | Glucose | Sodium Benzoate | Uric Acid | Creatinine | Urea | Xanthine (30µM) |
|---|---|---|---|---|---|---|---|Figure S10. Initial selectivity coefficients for common interferents (ascorbic acid (40 μM), glucose (100 μM), sodium benzoate (40 μM), uric acid (100 μM), creatinine (5 mM), urea (150 mM), and xanthine (30 μM) for the Pt / TEES (XOx) + MPC / PU (75:25) systems where TEES is doped with (A) C6 MPC, (B) C4 MPC, and (C) C3 MPC. Note: Legend at bottom applies to all three figures.
A
### Chart
| Category | Ascobic Acid | Glucose | Sodium Benzoate | Uric Acid | Creatinine | Urea | Xanthine (30µM) |
|---|---|---|---|---|---|---|---|B
### Chart
| Category | Ascobic Acid | Glucose | Sodium Benzoate | Uric Acid | Creatinine | Urea | Xanthine (30µM) |
|---|---|---|---|---|---|---|---|C
S13

## Slide 14
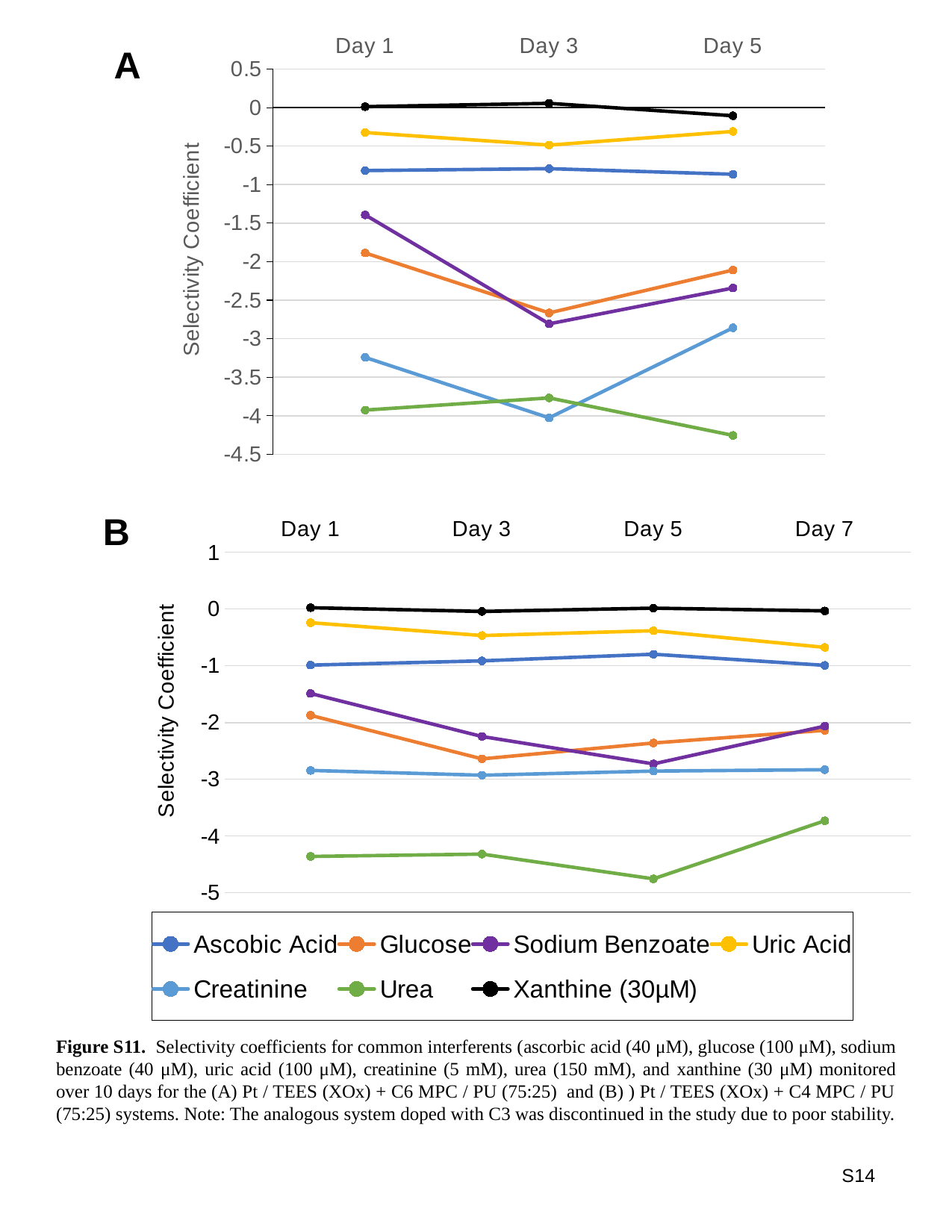

### Chart
| Category | Ascobic Acid | Glucose | Sodium Benzoate | Uric Acid | Creatinine | Urea | Xanthine (30µM) |
|---|---|---|---|---|---|---|---|
| Day 1 | -0.81767434 | -1.887677864 | -1.393316872 | -0.324209706 | -3.242986526 | -3.926132877 | 0.011493383 |
| Day 3 | -0.7929220329 | -2.665433673 | -2.807002624 | -0.4876067538 | -4.027077189 | -3.76819274 | 0.05441415864 |
| Day 5 | -0.865836595 | -2.108914088 | -2.342198911 | -0.3099099398 | -2.858330523 | -4.255034665 | -0.1060276112 |A
B
### Chart
| Category | Ascobic Acid | Glucose | Sodium Benzoate | Uric Acid | Creatinine | Urea | Xanthine (30µM) |
|---|---|---|---|---|---|---|---|
| Day 1 | -0.9872687048707529 | -1.87264176269219 | -1.4872695978426091 | -0.2395581101490301 | -2.843385057283407 | -4.360297456144204 | 0.024644617559589792 |
| Day 3 | -0.9120375796 | -2.639385152 | -2.245186423 | -0.4660218736 | -2.927713816 | -4.319252188 | -0.04185787341 |
| Day 5 | -0.795200802 | -2.359908176 | -2.728148275 | -0.3814585207 | -2.854517479 | -4.755068412 | 0.0163280999 |
| Day 7 | -0.9914900133 | -2.136464507 | -2.062004285 | -0.6751482236 | -2.830850533 | -3.730432715 | -0.03186347808 |Figure S11. Selectivity coefficients for common interferents (ascorbic acid (40 μM), glucose (100 μM), sodium benzoate (40 μM), uric acid (100 μM), creatinine (5 mM), urea (150 mM), and xanthine (30 μM) monitored over 10 days for the (A) Pt / TEES (XOx) + C6 MPC / PU (75:25) and (B) ) Pt / TEES (XOx) + C4 MPC / PU (75:25) systems. Note: The analogous system doped with C3 was discontinued in the study due to poor stability.
S14

## Slide 15
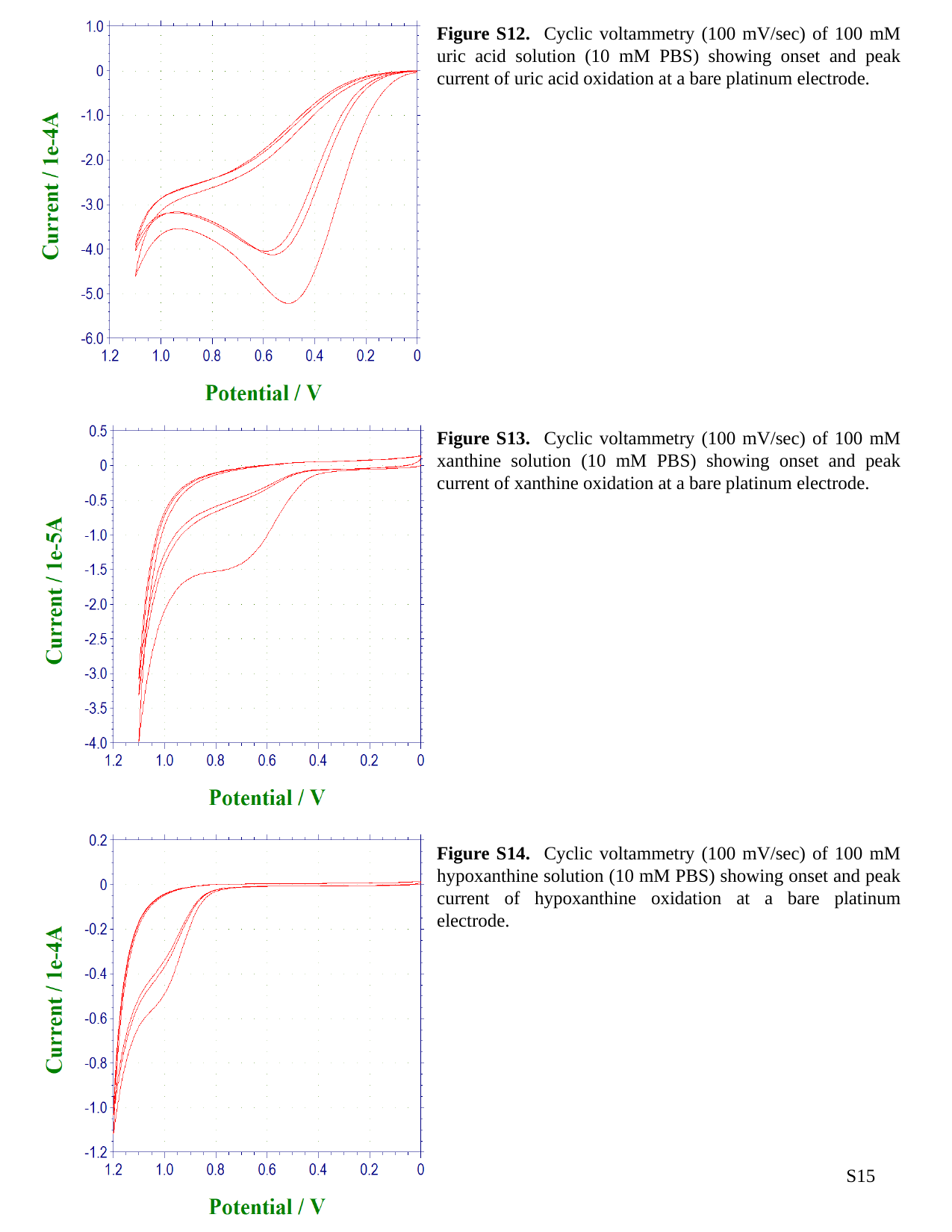

Figure S12. Cyclic voltammetry (100 mV/sec) of 100 mM uric acid solution (10 mM PBS) showing onset and peak current of uric acid oxidation at a bare platinum electrode.
Figure S13. Cyclic voltammetry (100 mV/sec) of 100 mM xanthine solution (10 mM PBS) showing onset and peak current of xanthine oxidation at a bare platinum electrode.
Figure S14. Cyclic voltammetry (100 mV/sec) of 100 mM hypoxanthine solution (10 mM PBS) showing onset and peak current of hypoxanthine oxidation at a bare platinum electrode.
S15

## Slide 16
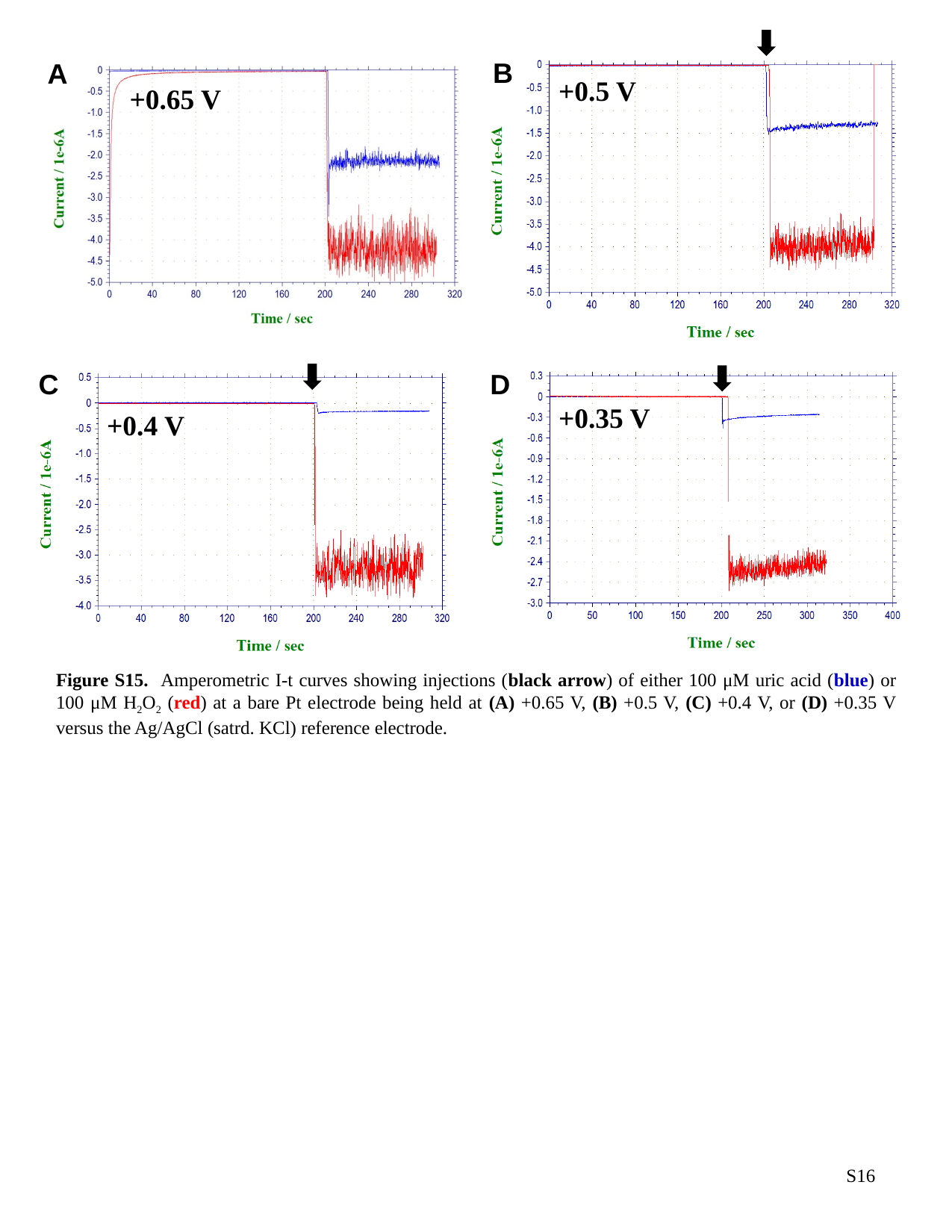

B
A
+0.5 V
+0.65 V
C
D
+0.35 V
+0.4 V
Figure S15. Amperometric I-t curves showing injections (black arrow) of either 100 μM uric acid (blue) or 100 μM H2O2 (red) at a bare Pt electrode being held at (A) +0.65 V, (B) +0.5 V, (C) +0.4 V, or (D) +0.35 V versus the Ag/AgCl (satrd. KCl) reference electrode.
S16

## Slide 17
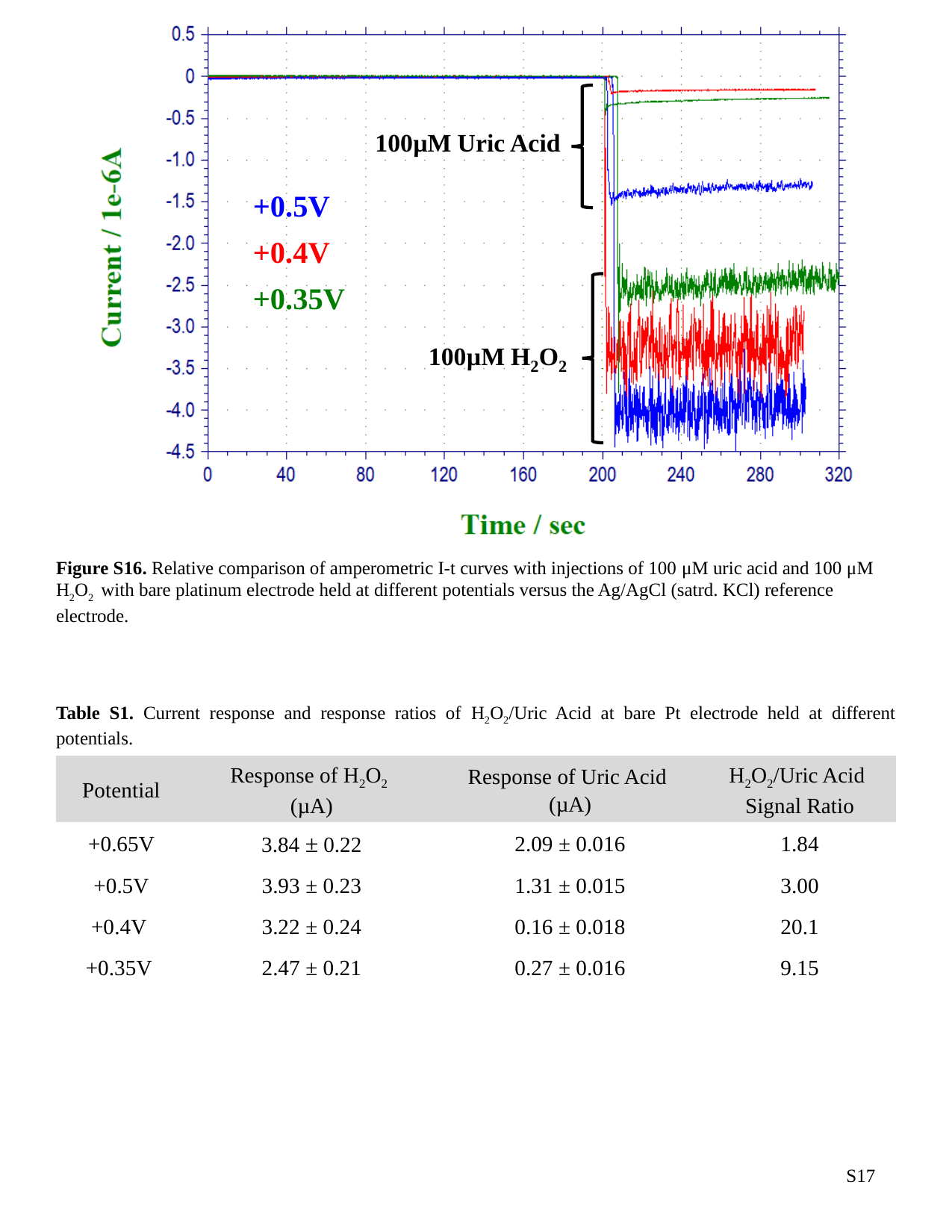

Figure S16. Relative comparison of amperometric I-t curves with injections of 100 μM uric acid and 100 μM H2O2 with bare platinum electrode held at different potentials versus the Ag/AgCl (satrd. KCl) reference electrode.
Table S1. Current response and response ratios of H2O2/Uric Acid at bare Pt electrode held at different potentials.
| Potential | Response of H2O2 (µA) | Response of Uric Acid (µA) | H2O2/Uric Acid Signal Ratio |
| --- | --- | --- | --- |
| +0.65V | 3.84 ± 0.22 | 2.09 ± 0.016 | 1.84 |
| +0.5V | 3.93 ± 0.23 | 1.31 ± 0.015 | 3.00 |
| +0.4V | 3.22 ± 0.24 | 0.16 ± 0.018 | 20.1 |
| +0.35V | 2.47 ± 0.21 | 0.27 ± 0.016 | 9.15 |
S17

## Slide 18
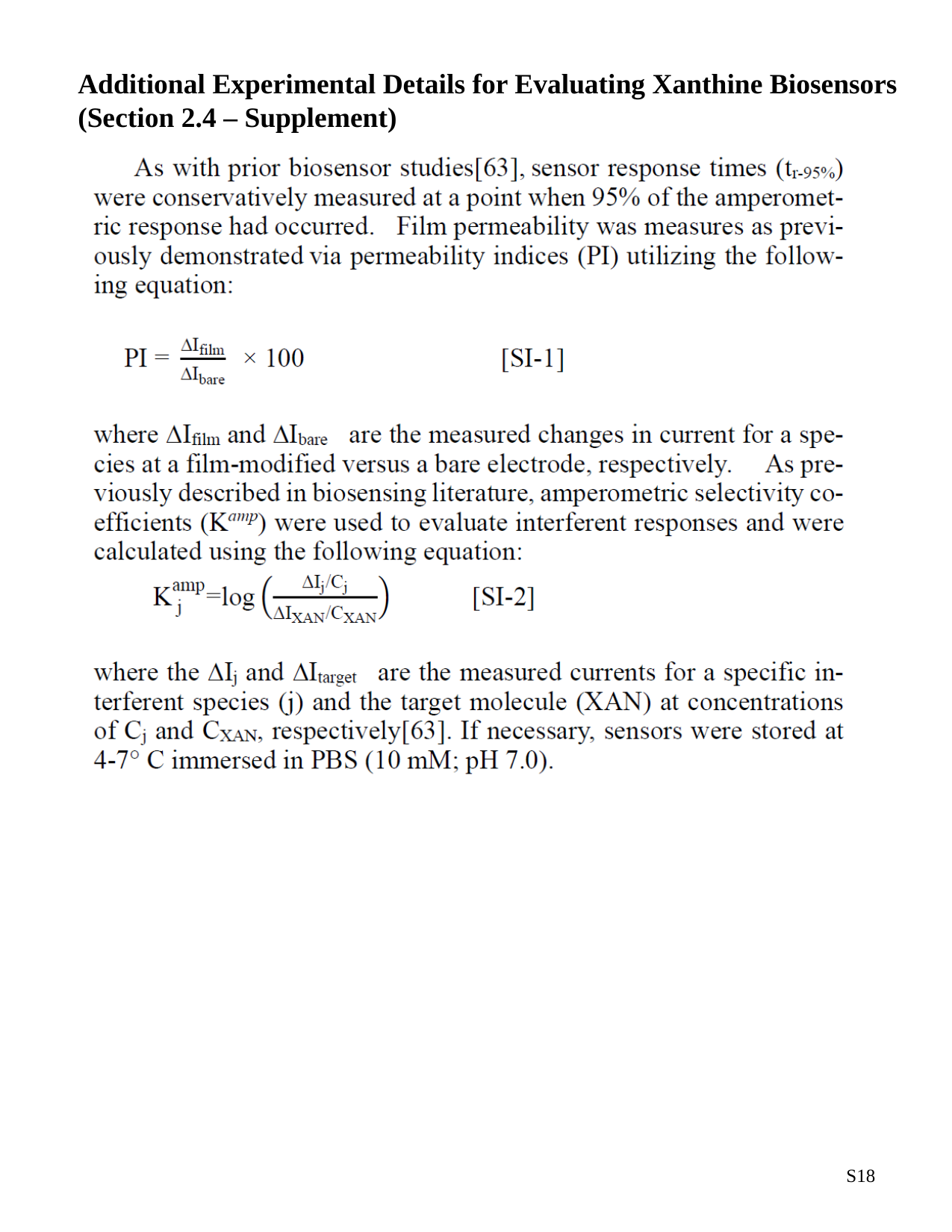

Additional Experimental Details for Evaluating Xanthine Biosensors
(Section 2.4 – Supplement)
S18
